# Control of protein synthesis through mRNA pseudouridylation by dyskerin

**DOI:** 10.1126/sciadv.adg1805

**Published:** 2023-07-28

**Authors:** Chiara Pederiva, Davide M. Trevisan, Dimitra Peirasmaki, Shan Chen, Sharon A. Savage, Ola Larsson, Jernej Ule, Laura Baranello, Federico Agostini, Marianne Farnebo

**Affiliations:** ^1^ Department of Cell and Molecular Biology, Karolinska Institutet, Solna 17165, Sweden.; ^2^ Department of Biosciences and Nutrition, Karolinska Institutet, Huddinge 14152, Sweden.; ^3^ Department of Oncology and Pathology, Karolinska Institutet, Solna 17165, Sweden.; ^4^ Science for Life Laboratory, Stockholm 17165, Sweden.; ^5^ Clinical Genetics Branch, Division of Cancer Epidemiology & Genetics, National Cancer Institute, National Institutes of Health, U.S. Department of Health and Human Services, Bethesda, MD 20852, USA.; ^6^ The Francis Crick Institute, London NW1 1AT, UK.; ^7^ UK Dementia Research Institute, King’s College London, London W1T 7NF, UK.; ^8^ National Institute of Chemistry, 1001 Ljubljana, Slovenia.; ^9^ Department of Medical Biochemistry and Biophysics, Karolinska Institutet, Solna 17165, Sweden.

## Abstract

Posttranscriptional modifications of mRNA have emerged as regulators of gene expression. Although pseudouridylation is the most abundant, its biological role remains poorly understood. Here, we demonstrate that the pseudouridine synthase dyskerin associates with RNA polymerase II, binds to thousands of mRNAs, and is responsible for their pseudouridylation, an action that occurs in chromatin and does not appear to require a guide RNA with full complementarity. In cells lacking dyskerin, mRNA pseudouridylation is reduced, while at the same time, de novo protein synthesis is enhanced, indicating that this modification interferes with translation. Accordingly, mRNAs with fewer pseudouridines due to knockdown of dyskerin are translated more efficiently. Moreover, mRNA pseudouridylation is severely reduced in patients with dyskeratosis congenita caused by inherited mutations in the gene encoding dyskerin (i.e., *DKC1*). Our findings demonstrate that pseudouridylation by dyskerin modulates mRNA translatability, with important implications for both normal development and disease.

## Teaser

Modification of uridines in mRNAs represents an intrinsic mechanism used by cells to control protein expression.

## INTRODUCTION

Pseudouridylation (the conversion of uridine into pseudouridine), the first found posttranscriptional modification of RNA (*1*, *2*), is now recognized as the most common. Pseudouridine can affect the structure and function of various noncoding RNAs (ncRNAs), including transfer RNA (tRNA), spliceosomal small nuclear RNA (snRNA), and ribosomal RNA (rRNA), influencing their interactions with proteins and other RNAs (*3*, *4*). Therefore, recent findings that pseudouridine is also present in mRNA (*5*–*10*) have raised the question as to how this modification might influence gene expression.

Pseudouridine in mRNA can affect its stability (*10*–*13*) and translation (*14*–*19*), and, when present in pre-mRNA, its splicing (*20*, *21*) and processing of the 3′ end (*21*). However, because most functional studies in this context involve mRNAs pseudouridylated in vitro, the role of endogenous pseudouridylation, as well as the enzyme(s) involved, remain largely unclear. In the case of pre-mRNAs, pseudouridylation was recently found to be catalyzed primarily by the pseudouridine synthase (PUS) enzymes PUS1, PUS7, and RNA PUS D4 (RPUSD4) (*21*). Although PUS1, PUS7, TruB PUS Family Member 1 (TRUB1), and dyskerin have been shown to pseudouridylate mature mRNA (*3*, *7*, *8*, *10*), removal of PUS1, PUS7, or TRUB1 does not alter the total level of pseudouridine in mRNA (*22*). This suggests functional redundancy among these enzymes and/or that additional enzymes that catalyze the same process remain to be identified.

In general, pseudouridylation is catalyzed by a class of enzymes known as PUS, of which 13 have been identified in humans (*3*). Dyskerin, the only PUS known to be guided by RNA, functions in close association with the GAR1, NOP10, and NHP2 proteins and box H/ACA guide RNAs (*23*). Together, these form the H/ACA complex, the RNA component of which identifies the nucleotide in the target RNA to be modified through sequence complementarity (*24*). Different classes of H/ACA RNAs, collectively named so because of the H/ACA motifs within their sequences, allow dyskerin to pseudouridylate different target RNAs. Small nucleolar H/ACA RNAs (snoRNAs) guide pseudouridylation of rRNA in the nucleolus, whereas H/ACA small Cajal body–associated RNAs (scaRNAs) target snRNA in Cajal bodies (*25*). Through its association with the telomerase RNA (which is also an H/ACA RNA), dyskerin also plays a critical role in telomere synthesis (*26*, *27*). Moreover, dyskerin binds to many nucleoplasmic Alu-derived AluACA and orphan H/ACA RNAs (*28*, *29*), which do not display complementarity with rRNA, snRNA, or telomeres, indicating the existence of additional substrates and sites of action for this enzyme.

The critical role of the H/ACA complex in cellular homeostasis is evident from the observation that inherited mutations in the genes encoding dyskerin [i.e., *Dyskeratosis Congenita 1* (*DKC1*)], NHP2 and NOP10, cause the telomere disorder known as dyskeratosis congenita and its more severe form, referred to as Hoyeraal-Hreidarsson syndrome, associated with various developmental defects, shorter telomeres, and an increased risk for cancer (*30*–*36*). Although these pathological features are primarily connected to impaired telomere elongation, defective RNA pseudouridylation is also believed to contribute, because mutations in the catalytic domain of dyskerin are associated with more severe disease (*37*). In addition, mice carrying mutant *Dkc1* exhibit perturbed pseudouridylation and develop this disease long before alterations in telomeres can be detected (*38*). Accordingly, defects in pseudouridylation due to mutations in *DKC1* and *NOP10* were recently shown to drive a nephrotic syndrome associated with early lethality (*39*). Moreover, *Dkc1*-deficient mice die in utero (*40*), while zebrafish with mutant *nop10* and *gar1* and morphant *dkc1* die early, all without any abnormal shortening of telomere length (*41*, *42*). Together, these observations indicate that disrupted pseudouridylation can result in severe pathological phenotypes.

Nuclear speckles, also known as splicing speckles or interchromatin granule clusters, were initially considered to be primarily storage sites for factors involved in pre-mRNA splicing (*43*). However, it is now evident that nuclear speckles are also enriched in proteins involved in transcription, mRNA modification and export, as well as in chromatin organization and localization, indicating that these domains are hubs for the integration of RNA polymerase II (RNAPII)–dependent transcription (*44*, *45*).

In the present investigation, on the basis of the observation that dyskerin is localized inside splicing speckles, we have examined the role of this enzyme and the H/ACA complex in connection with mRNA pseudouridylation and the consequences of this modification for gene expression. Using several high-throughput sequencing approaches, we show that the H/ACA complex is recruited to actively transcribed genes genome-wide, where it performs mRNA pseudouridylation. Moreover, we demonstrate that this modification reduces the rate of mRNA translation and that loss of dyskerin-mediated pseudouridylation results in global elevation of protein synthesis. Furthermore, this process is disrupted in patients with dyskeratosis congenita, suggesting that pseudouridylation of mRNA plays a role in the underlying pathogenesis. Consequently, we propose that cotranscriptional pseudouridylation of mRNA by the H/ACA complex enables coordination of transcription and translation.

## RESULTS

### The PUS dyskerin is localized to splicing speckles as part of the H/ACA complex

**Fig. 1.**
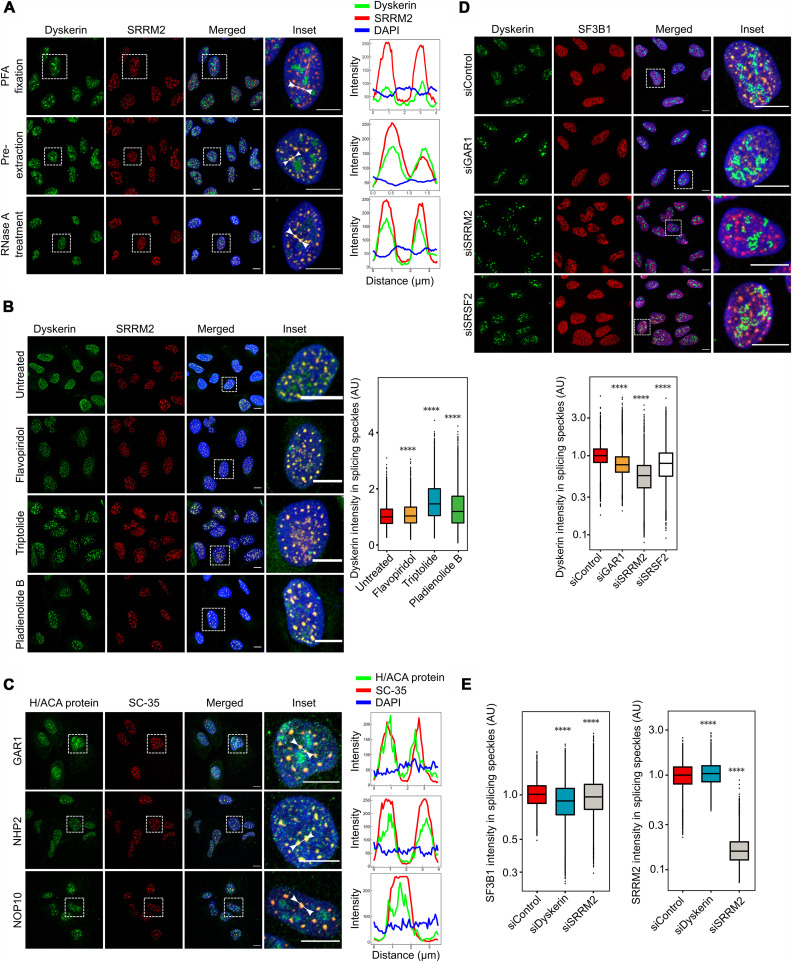
Dyskerin is localized in nuclear speckles. (**A**) U2OS cells immunostained for dyskerin (green, Abnova antibody) and the speckle protein SRRM2 (red). Paraformaldehyde (PFA) fixation indicates fixation with 4% formaldehyde followed by permeabilization; “Pre-extraction” indicates permeabilization of cells with cytoskeleton buffer 5 min before fixation with PFA; “RNase A” is the same as pre-extraction, but with addition of RNase A (300 μg/ml) to the cytoskeleton buffer. Intensity profiles were calculated above the speckles indicated between the two arrows. DAPI, 4′,6-diamidino-2-phenylindole. (**B**) U2OS cells treated with 250 nM flavopiridol, 1 μM triptolide, or 100 nM pladienolide B for 2 hours, followed by treatment with RNase A and immunostaining for dyskerin (green, Abnova antibody) and SRRM2 (red). Plots show the distribution of signal intensity of dyskerin inside nuclear speckles, as determined by CellProfiler, three independent experiments, >60 cells per experiment. AU, arbitrary units. (**C**) U2OS cells were treated with pladienolide B for 2 hours, then treated with RNase A, and immunostained for GAR1, NHP2, or NOP1 (green) and the speckle marker SC-35 (red). Intensity profiles were calculated above the indicated speckles. (**D**) U2OS cells treated with the small interfering RNA (siRNAs) indicated for 48 hours and immunostained for dyskerin (green, Abnova antibody) and SF3B1 (red). The plot below the images shows the distribution of signal intensity of dyskerin inside nuclear speckles, as determined by CellProfiler, five independent experiments, >60 cells per experiment. (**E**) U2OS cells treated with the siRNAs indicated for 48 hours and immunostained for SF3B1 or SRRM2. Plots show the distribution of their signal intensity inside nuclear speckles, as determined by CellProfiler, four independent experiments, >60 cells per experiment. ****P ≤ 0.0001, as determined by a Wilcoxon rank sum test. Scale bar (white line), 10 μm.

In addition to its established localization in nucleoli and Cajal bodies, as confirmed here by costaining with the marker proteins nucleolin and coilin, respectively (fig. S1A), dyskerin was also shown to localize to nuclear splicing speckles (Fig. 1A) by costaining with the marker protein serine arginine repetitive matrix 2 (SRRM2) (*46*). This localization was observed using different antibodies directed against dyskerin and in both transformed and nontransformed cells (fig. S1, B and C). Detection of dyskerin in splicing speckles was enhanced by removal of background signal through pre-extraction of soluble cellular proteins with detergent and even more so following removal of RNA by ribonuclease A (RNase A) before fixation (Fig. 1A). The latter finding indicates that localization of dyskerin to these nuclear subcompartments is independent of RNA, in contrast to its localization in nucleoli (Fig. 1A).

To explore the potential function of dyskerin in speckles, we first showed that accumulation of this enzyme in these structures is augmented by inhibition of RNAPII-mediated transcription (using flavopiridol or triptolide, which prevent transcriptional elongation and initiation, respectively) or splicing [using pladienolide B, which targets the splicing factor 3b subunit 1 (SF3B1) subunit of the spliceosome] (Fig. 1B). This result resembles the corresponding effects on factors known to be associated with transcription or splicing (*45*). Moreover, while GAR1, NOP10, and NHP2 could not be robustly detected in speckles in untreated cells (fig. S1D), inhibition of splicing allowed detection of these proteins in speckles (Fig. 1C), indicating that the entire H/ACA complex is present at this location. Consistently, knockdown of GAR1 reduced the intensity of the dyskerin signal in speckles, providing further support for collaboration between these two proteins at these sites (Fig. 1D and fig. S1E). Accumulation of dyskerin in speckles was also abrogated following depletion of the speckle markers SRRM2 and serine and arginine rich splicing factor 2 (SRSF2) (Fig. 1D and fig. S1E), both of which serve as molecular platforms for many proteins (*46*).

Next, we investigated the potential involvement of dyskerin in organizing the layered structure of splicing speckles (*47*). Depletion of dyskerin lowered the level of the peripheral region protein SF3B1 (a component of the spliceosome), but not that of the core protein SRRM2, which instead was slightly enhanced (Fig. 1E and fig. S1E), suggesting that dyskerin is involved in the retention of splicing factors at different layers of nuclear speckles. Together, these findings demonstrate that dyskerin is localized in splicing speckles as part of the mature H/ACA complex and that this localization is influenced by multiple factors related to transcription and splicing, indicating that the H/ACA holoenzyme is also involved in these processes.

### Dyskerin associates cotranscriptionally with chromatin and RNAPII

**Fig. 2.**
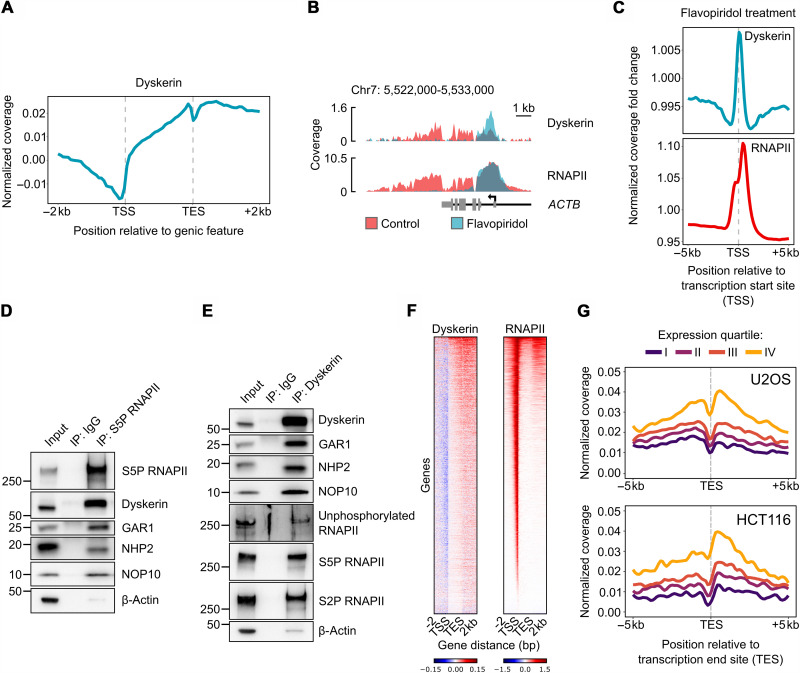
Dyskerin associates with active regions of chromatin through RNAPII. (**A**) Metadata profile of dyskerin ChIP-seq coverage over expressed [transcripts per million (TPM) > 1] protein-coding genes in U2OS cells (based on two independent experiments). The *x* axis shows scaled genomic regions, while the *y* axis shows the normalized log_2_ fold change compared to the input signal. (**B**) ChIP-seq profile of dyskerin and RNAPII (precipitated with antibody MAB0601) at the *ACTB* gene in control and flavopiridol-treated U2OS cells. The ChIP-seq signal is normalized to input and represents the average of two independent experiments. (**C**) Metadata profile of dyskerin and RNAPII ChIP-seq coverage around the TSS in U2OS cells after flavopiridol treatment (based on two independent experiments). The *y* axis shows the signal ratio between the flavopiridol treatment and the untreated control, as calculated after normalization of the signal over the corresponding inputs. (**D**) Immunoprecipitation (IP) of RNAPII or immunoglobulin G (IgG) (negative control) from the chromatin fraction of U2OS cells followed by Western blotting of the proteins indicated. A characteristic blot is shown. (**E**) IP of dyskerin or IgG from the chromatin fraction of U2OS cells followed by Western blotting of the proteins indicated. A characteristic blot is shown. (**F**) Heatmaps of dyskerin and RNAPII ChIP-seq signals along expressed (TPM > 1) protein-coding genes. Rows indicate all genes and are sorted by decreasing RNAPII occupancy. Signal abundances are displayed as colors ranging from blue (low binding) to red (high binding). (**G**) Metadata profile of dyskerin ChIP-seq coverage around the TES of expressed (TPM > 1) protein-coding genes in U2OS (top) and HCT116 (bottom) cells (based on two independent experiments). The *x* axis shows the distance to the TES, while the *y* axis shows the normalized log_2_ fold changes compared to the input signal. Expressed genes were divided into quartiles according to their TPM values (from three RNA-seq experiments).

Because nuclear speckles are closely associated with transcription by RNAPII (*45*), we subsequently examined the potential involvement of dyskerin in this process. Chromatin immunoprecipitation coupled to sequencing (i.e., ChIP-seq) showed genome-wide enrichment of dyskerin at expressed genes. At these sites, the levels of dyskerin were lowest at the transcription start site (TSS), increased along the gene body, and lastly peaked toward the transcription end site (TES) (Fig. 2A). Following inhibition of transcriptional elongation (using flavopiridol), dyskerin and RNAPII both accumulated around the TSS (Fig. 2, B and C), and after inhibition of transcriptional initiation (using triptolide), both disappeared from chromatin (fig. S2A). These observations are indicative of a functional relationship between these complexes and, moreover, suggest that dyskerin associates with RNAPII already at the beginning of genes.

To further characterize the connection between dyskerin and transcription, we found immunoprecipitation of RNAPII from cells to coprecipitate dyskerin, as well as NHP2, NOP10, and GAR1 (Fig. 2D). The interaction between RNAPII and dyskerin was partially disrupted by treatment with RNase A, suggesting that RNA facilitates or contributes to this association (fig. S2B). Reciprocal immunoprecipitation of dyskerin confirmed its binding to RNAPII and, in addition, suggested that it associates primarily with the hyperphosphorylated form of RNAPII (in which the residues serine 2 or 5 is modified) (Fig. 2E). Together, these results indicate that following initiation of transcription, dyskerin is recruited to genes by RNAPII and then travels along genes in association with the polymerase.

Ranking genes according to their level of RNAPII binding revealed a positive correlation between this binding and dyskerin occupancy (Fig. 2F). Consistently, in both U2OS and HCT116 cells, the binding of dyskerin to the TES of genes was proportional to their level of expression (Fig. 2G). There was also a strong correlation between the genes to which dyskerin was bound in these cell lines (fig. S2C), suggesting conservation of the function of dyskerin. Notably, the binding of dyskerin to transcribed genes was not dependent on the presence of a snoRNA sequence inside their introns (Fig. 2B and fig. S2, D and E), a previously established requirement for the association of dyskerin with a reporter gene (*48*). Together, these results demonstrate that dyskerin associates with RNAPII and is thereby enriched in genes transcribed by RNAPII throughout the genome.

### Steady-state levels of nuclear RNA are only mildly affected by depletion of the H/ACA complex

To gain insight into the function of dyskerin in connection with transcribed genes, we performed RNA sequencing (RNA-seq) expression profiling. Nuclear RNA was selected for this analysis to enhance detection of potential splicing defects and to match our individual-nucleotide resolution cross-linking and immunoprecipitation sequencing (iCLIP-seq) analysis (described in the next section). RNA from cells containing or depleted of dyskerin or GAR1 exhibited different patterns of spatial clustering (as revealed by principal components analysis), indicating that these different depletions affect RNA levels differently (fig. S3A). While knockdown of dyskerin significantly altered the expression of 135 genes, knockdown of GAR1 influenced the expression of 39 genes (fig. S3B). Of these, 11 were up-regulated by both knockdowns, whereas none were commonly down-regulated (fig. S3C).

As reported previously (*49*), 42 ncRNAs (mainly sno/scaRNAs) and two of their small nucleolar RNA host genes were among the transcripts down-regulated by depletion of dyskerin (fig. S3D), whereas the expression of none of these was affected by knockdown of GAR1 (fig. S3D). Although we cannot exclude a global reduction in the nuclear content of RNA following depletion of these proteins, these findings indicate that their relative levels are largely unchanged. Moreover, no major changes in splicing could be detected in cells lacking dyskerin or GAR1 (fig. S3E). Together, these data indicate that dyskerin and GAR1 are not involved in splicing or maintenance of steady-state nuclear RNA levels and rather that the H/ACA complex performs another function at transcribed genes.

### Dyskerin and GAR1 bind to thousands of mRNAs

**Fig. 3.**
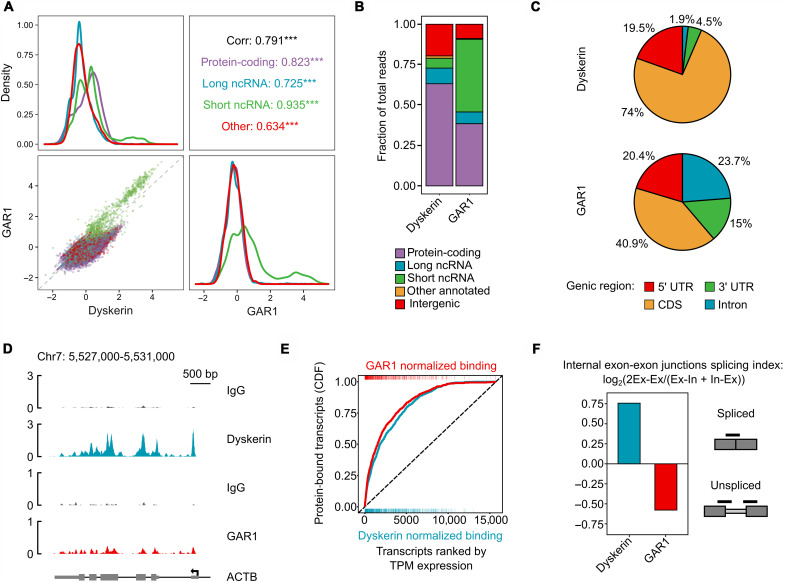
Dyskerin and GAR1 bind to mRNAs. (**A**) Correlation between iCLIP binding signals (log_10_) obtained from dyskerin or GAR1 in U2OS cells, divided by RNA biotype. The average of two independent experiments is shown. (**B**) Distribution of iCLIP reads from dyskerin and GAR1, divided by RNA biotype. The average of two independent experiments is shown. (**C**) Distribution of iCLIP peaks from dyskerin and GAR1 mapped inside protein-coding RNAs, normalized to the size of the following genic regions: untranslated regions (UTR), coding DNA sequences (CDS), and introns. The average of two independent experiments is shown. (**D**) Distribution of the iCLIP signal from dyskerin or GAR1 along the *ACTB* gene. The average of two independent experiments is shown. (**E**) Cumulative distribution of protein-coding RNAs bound by dyskerin (blue) and GAR1 (red) across protein-coding RNAs ranked according to their RNA-seq expression (TPM) (the highest to the lowest from left to right). (**F**) Splicing index calculated for protein-coding RNAs bound by dyskerin or GAR1.

To determine whether the function of H/ACA might involve RNA binding, intact U2OS cells were exposed to ultraviolet C (UV-C) light to cross-link direct contacts between proteins and RNAs; their nuclei were then fractionated and lysed, dyskerin and GAR1 immunoprecipitated from these nuclear fractions, and fragments of their cross-linked RNAs sequenced by iCLIP (*50*). As expected, both dyskerin and GAR1 bound to RNAs and did so to a similar extent (Fig. 3A). Both proteins coprecipitated the vast majority of snoRNAs and scaRNAs expressed (fig. S4, A and B), known partners of the H/ACA complex. In addition, a large proportion of the cross-linking was to protein-coding mRNAs (Fig. 3B), 64% of all reads in the case of dyskerin.

Characterization of the binding of dyskerin and GAR1 revealed the highest interaction within the coding sequences of genes (CDS), followed by 5′ untranslated region (5′UTR), 3′UTR, and lastly introns (Fig. 3, C and D). Within introns, most binding was in deep intronic regions, peaking 6 to 8 kb from the nearest exon (fig. S4C), and mapped to repetitive sequences identified primarily as long interspersed nuclear elements (LINEs) and Alu sequences (members of the family of short interspersed nuclear elements) (fig. S4, D and E). Because both dyskerin and GAR1 are bound to these repeat sequences, and Alu RNAs can form ribonucleoprotein (RNP) particles with H/ACA proteins (*28*), we speculate that these repeat sequences are partners of the H/ACA complex.

A more detailed examination did not reveal any preferred binding motif for dyskerin or GAR1 in coding regions, but both bound to mRNAs to an extent proportional to the level of expression (Fig. 3E), analogous to the extent of association of dyskerin with genes (Fig. 2G). Accordingly, binding of dyskerin and GAR1 to mRNA (as seen by iCLIP) and the corresponding gene sequence (as seen by dyskerin binding in ChIP-seq) was enriched (fig. S4F), suggesting that the H/ACA complex binds to mRNAs cotranscriptionally.

The binding of dyskerin and GAR1 to mRNAs was not dependent on the presence of intronic LINE/Alu RNAs or sno/scaRNAs (fig. S4G), indicating that this binding has a function other than incorporation of intronic ncRNAs into H/ACA RNP particles. Our observation that dyskerin cross-links more extensively to coding regions of mRNAs than GAR1, while binding to guide RNAs (short ncRNA) was extensive in both cases (fig. S4H), indicates that mRNAs may be substrates for dyskerin.

In light of the interactions of dyskerin and GAR1 with both introns and coding sequences, we examined whether these interactions occur preferentially to nascent pre-mRNA or the mature form. Calculation of the splicing index [i.e., the ratio between interactions spanning exon-exon junctions and those including exon-intron junctions (*51*)] revealed more extensive binding of dyskerin to spliced RNAs (Fig. 3F). Although GAR1 appeared to bind primarily to precursor RNAs, exon-exon binding was substantially lower for GAR1 than for dyskerin, suggesting less cross-linking of GAR1 to spliced mRNAs rather than preferred binding to precursor RNA. Collectively, these results demonstrate that dyskerin and GAR1 interact directly with thousands of nuclear mRNAs. This association appears to have two purposes: binding of intronic Alu and LINE sequences to serve as potential guide RNAs and binding of coding mRNAs, probably after splicing, as substrates.

### Dyskerin pseudouridylates mRNA during transcription and this process is defective in dyskeratosis congenita associated with mutation of dyskerin

**Fig. 4.**
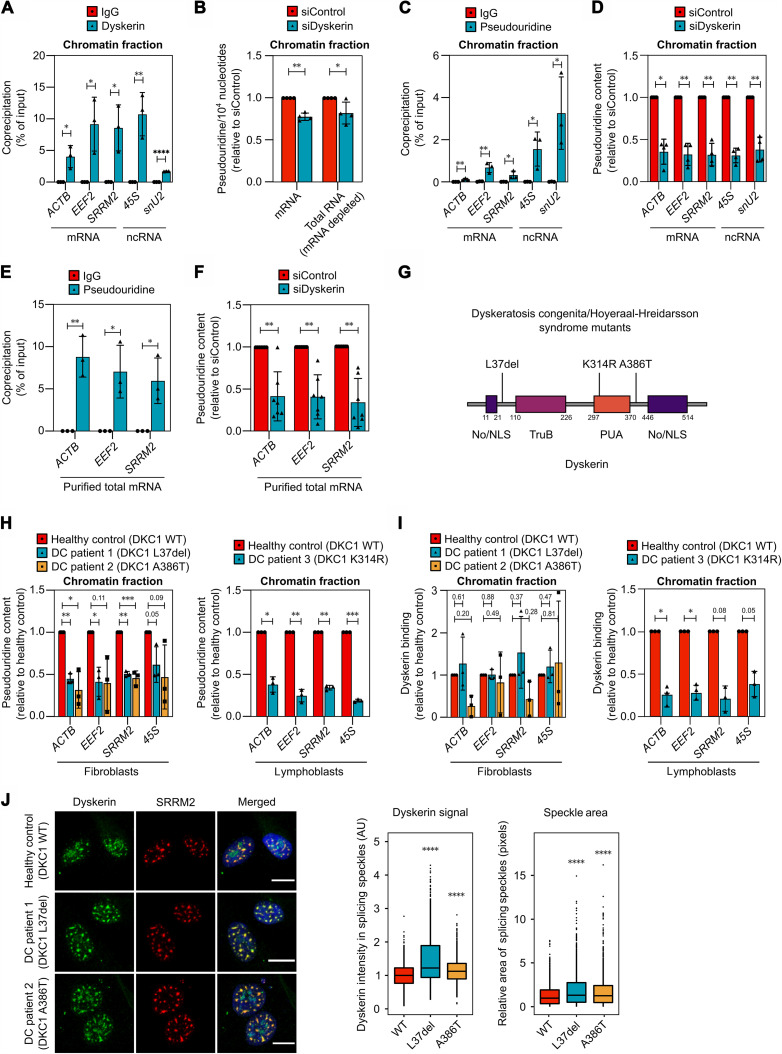
Dyskerin pseudouridylates mRNA cotranscriptionally and this process is disrupted in patients with dyskeratosis congenita. (**A**) RIP using an antibody targeting dyskerin from the chromatin fraction of U2OS cells. Shown is the amount of coprecipitated RNA as percentage of input (mean ± SD, three independent experiments) measured by qPCR. (**B**) Quantification of RNA modifications using LC-MS/MS. mRNA and total RNA remaining after mRNA depletion were extracted from chromatin fractions of U2OS cells treated with siRNA for 48 hours. Shown are the levels of pseudouridine normalized to the number of canonical ribonucleosides and relative to the control sample (mean ± SD, four independent experiments). (**C** and **D**) RIP using an antibody targeting pseudouridine from the chromatin fraction of U2OS cells. In (D), cells were treated with siRNA for 48 hours. (**E** and **F**) RIP using an antibody targeting pseudouridine from mRNA purified from whole U2OS cells. In (F), cells were treated with siRNA for 48 hours. (**G**) Dyskerin protein domains and the dyskeratosis congenita–associated mutations in the patient cells examined. No/NLS, nucleolar/nuclear localization signal; TruB, PUS domain; PUA, PUS and archaeosine transglycosylase domain. (**H** and **I**) RIP using an antibody targeting pseudouridine (H) or dyskerin (I) from the chromatin fraction of fibroblasts or lymphoblasts from patients with dyskeratosis congenita (DC) and healthy donors. (**J**) Fibroblasts from patients with DC and healthy donors were immunostained for dyskerin (green, Abnova antibody) and the speckle protein SRRM2 (red). Scale bar (white line), 10 μm. Plots show the distribution of dyskerin signal intensity inside nuclear speckles and area of speckle as determined by CellProfiler. Wilcoxon rank sum test. Two independent experiments, >30 cells per experiment. For RIP experiments, graphs show the coprecipitated RNA as percentage of input (mean ± SD, ≥3 independent experiments) measured by qPCR. RIP samples were compared as indicated using an unpaired two-tailed *t* test (A), (C), and (E), paired ratio two-tailed *t* test (I), or paired ratio one-tailed *t* test (B), (D), (F), and (H). *P < 0.05, **P < 0.01, ***P < 0.001, and ****P ≤ 0.0001.

To determine whether dyskerin and the H/ACA complex are involved in mRNA pseudouridylation, we first verified binding of this enzyme to various mRNAs (*ACTB*, *EEF2*, and *SRRM2*) and ncRNAs (*45S* rRNA and *U2* snRNA) identified by iCLIP using RNA immunoprecipitation (RIP). Dyskerin coprecipitated all RNAs examined from isolated chromatin (Fig. 4A), further supporting our proposal that mRNAs are bound by H/ACA cotranscriptionally.

To assess mRNA pseudouridylation, we first digested the RNA entirely and quantified global mRNA pseudouridylation using liquid chromatography–tandem mass spectrometry (LC-MS/MS). This revealed a reduction of this modification in mRNA isolated from chromatin following dyskerin knockdown, whereas other RNA modifications, including m6A or m7G, were not decreased (Fig. 4B and table S1). Moreover, pseudouridylation of the remaining RNA (depleted of mRNA), which consists primarily of rRNA, was also reduced (Fig. 4B), as expected.

To obtain a more detailed understanding of this process, including whether pseudouridylation of the mRNAs bound by dyskerin was affected, we performed RIP using an antibody against pseudouridine. The specificity of this commercially available antibody was first validated by immunoprecipitation of in vitro–transcribed *GFP* mRNA containing only uridine or pseudouridine, demonstrating specific recognition only of the pseudouridine-containing transcript (fig. S5A), in accordance with a previous report (*22*). Next, the antibody against pseudouridine was used for immunoprecipitation of endogenous RNAs, revealing coprecipitation of all RNAs associated with dyskerin in isolated chromatin, albeit to different extents (Fig. 4C). In agreement with the hypothesis that dyskerin catalyzes this pseudouridylation, immunoprecipitation with this specific antibody was reduced by 65% in cells depleted of dyskerin (Fig. 4D). Pseudouridylated *GFP* mRNA spiked into our samples before RIP with the antibody against pseudouridine was coprecipitated in a similar manner in all samples (fig. S5B), excluding the possibility that the reduction in mRNA pseudouridylation following loss of dyskerin is due to less efficient RNA-IP.

Subsequent immunoprecipitation of pseudouridylated RNA from purified mRNA rather than from total chromatin-associated RNA, as above, verified both the presence of pseudouridine in mRNA and the lower extent of this modification in cells lacking dyskerin (Fig. 4, E and F). Notably, following depletion of dyskerin, the amounts of pseudouridine in the mRNAs, rRNAs, and snRNAs examined were lowered to similar extents (Fig. 4, D and F), indicating that dyskerin plays a key role not only in the pseudouridylation of rRNA and snRNA, as previously shown, but also in introducing the same modification into mRNA.

Inherited mutations in dyskerin, resulting in defective rRNA pseudouridylation, are associated with the X-linked syndrome dyskeratosis congenita (*6*, *52*, *53*). To determine whether these disease-causing mutations in dyskerin result in similar defects in mRNA pseudouridylation, we examined fibroblasts and lymphoblasts from patients with dyskeratosis congenita harboring mutations in three different regions of this protein, namely, L37del, K314R, and A386T (Fig. 4G). Coprecipitation analysis of fibroblasts and lymphoblasts from healthy donors confirmed that dyskerin interacts with mRNA and rRNA (fig. S5C) and that these RNAs contain pseudouridines in both these cell types (fig. S5D). Analogous immunoprecipitation using cells from patients revealed lowered levels of pseudouridylation in rRNA (Fig. 4H). Moreover, the degree of mRNA pseudouridylation was also significantly attenuated in patient cells (Fig. 4H), to an extent similar to the reduction of pseudouridines in rRNA and as obtained following depletion of dyskerin (Fig. 4, D and F). These observations provide further support for the considerable impact of dyskerin on mRNA modification.

RIP revealed reduced binding of the K314R mutant of dyskerin to RNA (Fig. 4I), which is not unexpected, because this mutation is in the PUA RNA-binding domain of the enzyme. While the L37del and A386T variants still bound RNA to varying extents (Fig. 4I), they accumulated in nuclear splicing speckles to a larger extent than the wild-type protein (Fig. 4J) and, furthermore, these speckles were bigger in the cells from patients (Fig. 4J). We speculate that mutant dyskerin may be sequestered to a greater degree in splicing speckles due to the accumulation of unprocessed RNA, which may also increase speckle size (*47*). Together, these findings demonstrate that dyskerin is involved in mRNA pseudouridylation, a process that appears to occur cotranscriptionally, be conserved across different tissues and, importantly, to be defective in dyskerin-mutant forms of dyskeratosis congenita.

### mRNA pseudouridylation by dyskerin does not require a guide RNA with perfect complementarity

**Fig. 5.**
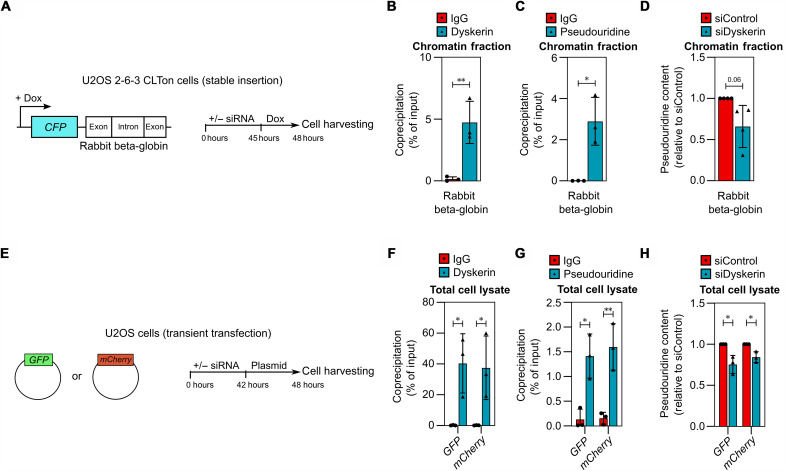
mRNA pseudouridylation by dyskerin does not appear to require a guide RNA with perfect complementarity. (**A**) Schematic representation of the U2OS 2-6-3 CLTon reporter system and experimental setup. (**B** to **D**) RIP using an antibody targeting dyskerin (B) or pseudouridine (C) and (D) from the chromatin fraction of U2OS 2-6-3 CLTon cells treated with doxycycline for 3 hours to induce the expression of the exogenous cassette. In (D), cells were treated with siRNA for 48 hours. The graphs show the amount of coprecipitated RNA as percentage of input (mean ± SD, ≥3 independent experiments) measured by qPCR. (**E**) Schematic representation of the GFP/mCherry reporter systems and experimental setup. (**F** to **H**) RIP using an antibody targeting dyskerin (F) or pseudouridine (G) and (H) from whole-cell lysate of U2OS cells transfected with plasmids encoding *GFP* or *mCherry* for 6 hours. In (H), cells were treated with siRNA for 48 hours. The graphs show the coprecipitated RNA as percentage of input (mean ± SD, ≥3 independent experiments) measured by qPCR. RIP samples were compared as indicated using an unpaired two-tailed *t* test (B), (C), (F), and (G) or a paired ratio one-tailed *t* test (D) and (H). *P < 0.05 and **P < 0.01.

Because pseudouridylation of rRNA and snRNA by dyskerin has been shown to involve sequence-specific guide RNAs, we next investigated whether such complementary guide RNAs are also required for mRNA pseudouridylation. For this purpose, we analyzed pseudouridylation of a nonhuman mRNA transcribed from an inducible cassette integrated stably into U2OS cells [U2OS 2-6-3 CLTon cells (*54*, *55*), Fig. 5A]. Because the reporter mRNA consists of a fusion between the coding sequence for a cyan fluorescent protein (CFP) and an exon-intron module from the rabbit beta-globin gene (*HBB2*), we assume that no guide RNA with perfect complementary is present within these cells. As was the case for endogenous mRNA, this exogenous transcript was coprecipitated with dyskerin (Fig. 5B), further indicating that the H/ACA complex interacts with any newly transcribed mRNA. This interaction occurred in chromatin fractions and, moreover, appeared to result in pseudouridylation, because in chromatin fractions the reporter mRNA was immunoprecipitated with the antibody targeting pseudouridine (Fig. 5C) and this immunoprecipitation was reduced in the absence of dyskerin (Fig. 5D).

Other exogenous mRNAs encoding green fluorescent protein (GFP) and monomeric (m)Cherry, produced from plasmids transiently transfected into cells (Fig. 5E), were also bound by dyskerin and pseudouridylated in a manner mediated at least in part by this enzyme (Fig. 5, F to H). This result provides further evidence that dyskerin can pseudouridylate transcripts produced from exogenous genes for which there should be no endogenous guide RNA with perfect complementary. The levels of these exogenous mRNAs were elevated in cells depleted of dyskerin (fig. S6, A and B), in contrast to the levels of most endogenous mRNAs, which remained relatively constant under the same conditions. The reason for this difference remains unclear. Together, our findings demonstrate that dyskerin associates with and can pseudouridylate both endogenous and exogenous mRNA without the involvement of a guide RNA with perfect complementarity. However, because the H/ACA complex cannot attain maturity and functionality without being bound to a guide RNA (*56*), mRNA pseudouridylation is probably achieved with the aid of guide RNAs containing mismatches toward the mRNA to be modified.

### mRNA pseudouridylation by dyskerin represses translation

**Fig. 6.**
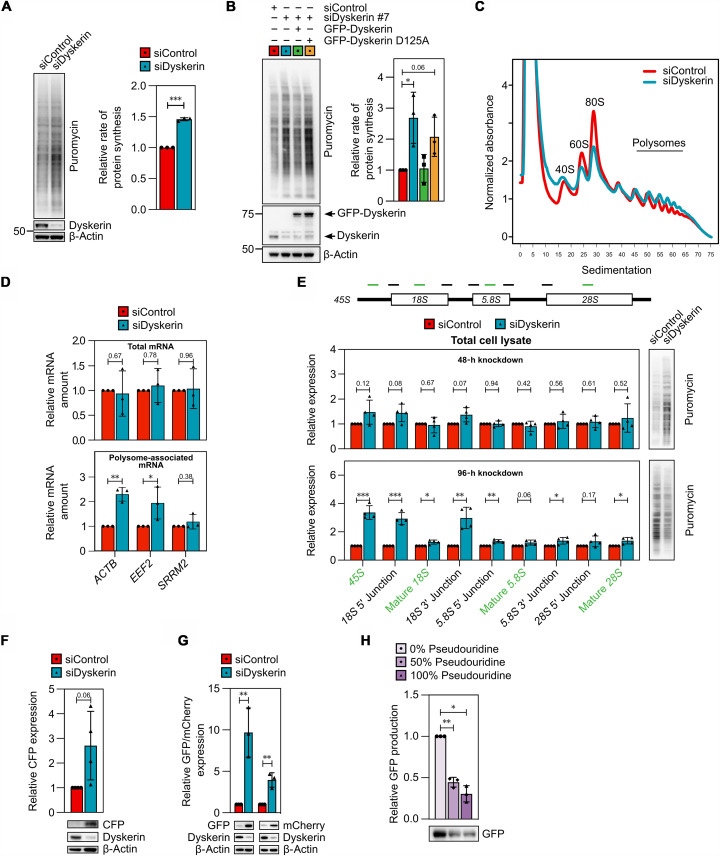
Loss of dyskerin-mediated mRNA pseudouridylation enhances protein synthesis. (**A** and **B**) U2OS cells transfected with siRNA for 48 hours (A) or for the last 16 hours also with plasmids encoding GFP-Dyskerin resistant to siDyskerin #7 (B), pulsed with puromycin, and analyzed by Western blot. Shown are a representative blot and quantification of puromycin normalized to β-actin and relative to siControl (mean ± SD, three independent experiments). (**C**) Polysomal fractionation of MCF7 cells treated with siRNA for 48 hours. Shown is a representative profile (three independent experiments). (**D**) Total mRNA and polysome-associated mRNA (associated with more than three ribosomes) from (C) were quantified using qPCR. Expression, relative to the siControl sample, is shown (mean ± SD, three independent experiments). (**E**) The expression of rRNA intermediates (left) and puromycin incorporation (right) in siRNA-treated U2OS cells analyzed by qPCR or Western blot, respectively. RNA levels are shown normalized to β-actin and relative to siControl (mean ± SD, four independent experiments). Full Western blots and quantifications are shown in fig. S7 (C and D). (**F**) U2OS 2-6-3 CLTon cells were treated with siRNA for 48 hours and for the last 3 hours with doxycycline and analyzed by Western blot. Shown are representative blots and densitometric quantification normalized as in (A) (four biological replicates). (**G**) U2OS cells were treated with siRNA for 48 hours and for the last 6 hours with plasmids encoding *GFP* or *mCherry* and analyzed by Western blot. Shown are representative blots and densitometric quantification normalized as in (A) (three biological replicates). (**H**) *GFP* mRNAs containing the indicated amount of pseudouridine were translated in wheat germ extract and protein production assessed by Western blotting. Shown is a representative blot and quantification normalized to the 0% pseudouridine sample (mean ± SD, three independent experiments). Samples were compared using a paired ratio two-tailed *t* test. *P < 0.05, **P < 0.01, and ****P ≤ 0.0001.

Because pseudouridylation can modulate the translatability of mRNA (*14*), we hypothesized that dyskerin may regulate mRNA translation. To test this, we measured protein synthesis using incorporation of puromycin in cells depleted from dyskerin. The rate of de novo protein synthesis in these cells was markedly elevated (Fig. 6A and fig. S7A), a phenomenon also observed in MCF7, HCT116, and WI-38 cells (fig. S7B), suggesting that this effect is not cell-type specific. This increase in translation was restored to control levels by small interfering RNA (siRNA)–resistant dyskerin (GFP-Dyskerin; Fig. 6B), which argues against off-targeting effects of the siRNA. Furthermore, a catalytically inactive form of dyskerin (GFP-Dyskerin D125A) failed to reverse the enhanced protein synthesis in dyskerin-depleted cells (Fig. 6B), reinforcing the conclusion that defective pseudouridylation drives the enhanced translational efficiency observed.

Polysome fractionation gave an analogous outcome, with elevated amounts of polysome-associated mRNA and a concomitant decrease in the 80S peak in cells with reduced expression of dyskerin (Fig. 6C), in accordance with a previous report (*57*). Notably, the mRNAs that associated to a greater extent with polysomes in cells depleted of dyskerin (Fig. 6D) included those previously shown to bind this protein (Fig. 4A) and whose levels of pseudouridine were significantly lowered (Fig. 4, C and E), further supporting regulation of mRNA translation through pseudouridylation by dyskerin. However, depletion of dyskerin did not alter translation of *SRRM2* mRNA, even though it contained fewer pseudouridines, indicating that other factors are also involved, at least in this case.

Considering the established role of dyskerin in rRNA processing (*58*–*61*), effects on rRNA maturation following knockdown of this protein may also potentially affect translation (*62*). To examine this possibility, nine polymerase chain reaction (PCR) primer pairs were used to characterize the synthesis and processing of rRNA (Fig. 6E). Although no significant alterations were detected following 48 hours of dyskerin knockdown, after 96 hours, the levels of precursor rRNA and several of its intermediates were elevated threefold (Fig. 6E). Such accumulation is indicative of disrupted rRNA processing, which impairs ribosome biogenesis (*63*). Accordingly, in contrast to the increase in protein synthesis observed 48 hours after reduction of dyskerin expression, longer depletion was associated with lowered incorporation of puromycin (Fig. 6E and fig. S7D). Consistently, reduced protein synthesis was observed in fibroblasts and lymphoblasts derived from patients with dyskeratosis congenita (fig. S7E). This alteration occurred without changes in phosphorylation of the key eukaryotic translation initiation factor eIF2α (fig. S7, C and D). Together these findings demonstrate that prolonged deficiency of dyskerin leads to defective rRNA processing, which in turn attenuates protein synthesis. In contrast, with dyskerin depletion of shorter duration, when rRNA processing is still intact, protein synthesis is enhanced, suggesting that this effect is not due to alterations in rRNA pseudouridylation but rather to defective mRNA pseudouridylation.

To examine the relationship between mRNA pseudouridylation and translation in greater detail, we depleted dyskerin for 48 hours and examined production of the CFP protein derived from the reporter mRNA previously shown to be pseudouridylated by dyskerin (Fig. 5D). Notably, 3 hours after induction of this exogenous gene, when the protein encoded was barely detectable in control cells, its protein level was threefold higher in cells depleted of dyskerin (Fig. 6F) and, at the same time, pseudouridylation of *CFP* mRNA in these cells was reduced (Fig. 5D). Similarly, following transient expression of GFP or mCherry, the levels of these proteins were approximately 10 and 4 times higher, respectively, in cells lacking dyskerin than in control cells (Fig. 6G), with less pseudouridine in their mRNAs (Fig. 5H). This enhancement was most apparent at early time points following protein induction, possibly due to the initiation of compensatory mechanisms at later time points (fig. S7F). Therefore, reduced mRNA pseudouridylation by dyskerin appears to enhance the translation of these exogenous proteins.

**Fig. 7.**
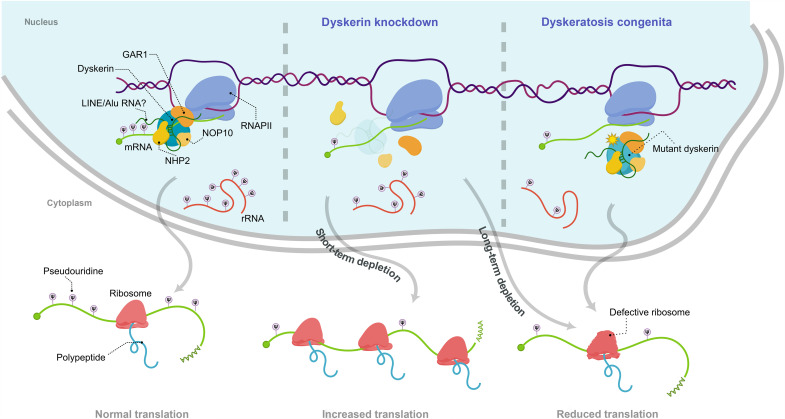
Schematic model of dyskerin-mediated mRNA pseudouridylation and protein synthesis. (Step 1) Dyskerin and the H/ACA complex associate with chromatin and RNAPII cotranscriptionally. (Step 2) This allows pseudouridylation of mRNA by dyskerin, potentially using LINE/Alu RNA as guides. In addition, dyskerin pseudouridylates rRNA in the nucleolus. (Step 3) Pseudouridines in mRNA reduce its translation allowing controlled protein synthesis. When the function of dyskerin is impaired, it has different outcomes on translation depending on the duration of impairment; short-term (48 hours) depletion enhances translation due to reduced mRNA pseudouridylation, while long-term (>96 hours) depletion attenuates translation due to defective rRNA pseudouridylation and processing. Inherited mutation of dyskerin in dyskeratosis congenita has a similar outcome on translation as long-term depletion of the enzyme.

To examine directly whether mRNA containing pseudouridine alters translation, we generated *GFP* mRNAs in vitro containing different proportions of pseudouridine. Because the RNA-dependent protein kinase (PKR) is activated by exogenous transcripts containing uridine and then phosphorylates eIF2α, thereby inhibiting translation initiation (*19*), wheat germ extracts lacking PKR were used in these experiments. Increased incorporation of pseudouridine led to progressive attenuation of the production of GFP (Fig. 6H), suggesting inhibition of translation by this modification, in accordance with previous reports (*15*). Together, these observations demonstrate that in situations where rRNA processing remains intact, dyskerin attenuates translational efficiency to an extent dependent on its ability to catalyze pseudouridylation. Considered together with our other findings that dyskerin can pseudouridylate mRNAs, which alters their association with polysomes and attenuates translation, at least in vitro, we conclude that cotranscriptional pseudouridylation of mRNAs by dyskerin plays a major role in their translation (Fig. 7).

## DISCUSSION

Here, we demonstrate that the PUS dyskerin, a component of the H/ACA complex, plays a major role in mRNA pseudouridylation, which in turn exerts a strong impact on mRNA translation in human cells. We show that dyskerin introduces pseudouridine into mRNAs already in chromatin, likely during their transcription, a process regulated by the recruitment of dyskerin to active genes by RNAPII, which in turn reduces translation of the corresponding mRNA (Fig. 7). On the basis of these findings, we propose that cotranscriptional pseudouridylation of mRNAs by the H/ACA complex represents an intrinsic mechanism used by cells to control protein expression.

Dyskerin and the H/ACA complex localize to Cajal bodies and nucleoli to pseudouridylate snRNA and rRNA, respectively (*25*). Our detection of dyskerin and the H/ACA complex in nuclear splicing speckles involved in mRNA processing (*45*) raised the possibility that this complex could also be involved in pseudouridylation of mRNA. Accordingly, we found that the H/ACA complex associates with RNAPII and travels along actively transcribed genes together with the polymerase. This phenomenon occurs throughout the genome, in different cell types, and is controlled by the activity of RNAPII. Thus, stalling or release of RNAPII on/from chromatin following inhibition of transcriptional elongation or initiation results in simultaneous stalling and release of dyskerin. Whether the localization of dyskerin in speckles reflects association with RNAPII and genes remains unclear. However, this localization does not appear to be essential, because it is enhanced by triptolide and observed more clearly after treatment with RNase A, and these same factors reduce the association of dyskerin with genes and RNAPII, respectively.

The involvement of the H/ACA complex in mRNA-related processes was further strengthened by our finding that dyskerin and GAR1 bind extensively to mRNA within the nucleus, both to introns and exons of genes that encode proteins. While the binding to introns occurs at LINE and Alu repeat sequences, no specific binding motifs could be identified in exons. However, dyskerin showed a strong preference for binding to spliced mRNAs expressed at high levels. This binding was independent of the presence of a sno/sca/LINE/Alu RNA sequence in the introns of their genes.

Moreover, dyskerin was more closely associated with the coding regions of mRNA than GAR1, highlighting these regions as targets, rather than partners of the H/ACA complex. Accordingly, in cells depleted of dyskerin, the amount of pseudouridine in mRNAs was reduced to less than half, indicating that dyskerin is responsible for a large proportion of mRNA pseudouridylation. While a 22% reduction in the number of pseudouridines in mRNA following dyskerin knockdown was detected with LC-MS/MS, usage of the antibody against pseudouridine indicated that this reduction was as much as 65%. This discrepancy might reflect the fact that mass spectrometry, although providing highly specific and sensitive quantification of this RNA modification, cannot discriminate its origin. Because the level of pseudouridines in, for example, tRNA, rRNA, and snRNA is 100-fold higher than in mRNA [(*3*); table S1], even minor contamination with other RNAs will interfere with determination of mRNA pseudouridylation and give a background signal that may mask a specific reduction of pseudouridine in mRNA. Moreover, LC-MS/MS quantified pseudouridine globally in all mRNAs, probably including mRNAs potentially targeted primarily by one or more of the 12 additional PUS enzymes other than the H/ACA complex. On the other hand, RIP with the antibody against pseudouridine detected mRNAs strongly bound to dyskerin in iCLIP and thereby potentially modified preferentially by dyskerin and thus more susceptible to knockdown of this enzyme. It was recently reported that dyskerin pseudouridylates hundreds of synthetic mRNAs whose sequences are to some degree complementary to those of endogenous snoRNAs (*64*) and that the amounts of pseudouridine in these synthetic mRNAs also lowered approximately 60% following knockdown of dyskerin, which is consistent with our current data. Furthermore, the connection between dyskerin and certain pseudouridine residues in endogenous mRNA (*6*, *10*, *11*), as well as the interaction of dyskerin with cytoplasmic transcripts (*57*), support even more an involvement of dyskerin in mRNA pseudouridylation. Still, determining the functional consequences of this modification of additional and individual mRNAs is required to understand this form of regulation in greater detail.

The fact that modification of rRNA and snRNA by dyskerin involves sequence-specific guide RNAs (*23*, *65*) raises the question as to whether this is also the case with pseudouridylation of mRNA. Our finding that dyskerin can pseudouridylate in principle any mRNA, including those transcribed from exogenous genes introduced into cells only a few hours earlier, argues against a requirement for pre-existing guides with perfect complementarity. Although perfect complementarity does not seem to be essential, a guide RNA is likely involved as the H/ACA complex cannot reach maturity and functionality without binding a guide RNA (*56*). Several studies have shown that the H/ACA complex can still pseudouridylate when there are a certain number of mismatches between its guide and substrate RNA, but more slowly (*24*, *66*–*69*). Although snoRNAs can guide pseudouridylation of synthetic mRNA, the extent to which this occurs in vivo would appear to be limited by the localization of snoRNAs in the nucleolus and mRNA in the nucleoplasm (*64*).

Our observation that dyskerin and GAR1 bind abundantly to many different intronic Alu and LINE repeat sequences makes them potential candidates for assisting pseudouridylation of mRNA. These repeat RNAs are localized in the nucleoplasm and chromatin (*28*, *70*). Moreover, RNA containing intronic Alu repeats can be processed into RNAs that are structurally highly reminiscent of box H/ACA RNAs and can form metabolically stable AluACA complexes with H/ACA proteins (*28*, *71*). The broad sequence variability of the putative pseudouridylation loop of AluACA RNAs further indicates that these can direct pseudouridylation of many different target RNAs.

A remaining key question involves the functional consequences of mRNA pseudouridylation by the H/ACA complex. Posttranscriptional mRNA modifications can influence several aspects of the mRNA life cycle, including stability, splicing, structure, localization and translation (*72*). Our finding that the level of nuclear mRNA in cells depleted of dyskerin or GAR1 was largely unchanged argues against the involvement of dyskerin and associated pseudouridylation in regulating mRNA stability. Splicing also appeared to be normal in cells lacking dyskerin or GAR1, which is consistent with our observation that dyskerin binds more strongly to spliced mRNAs, indicating that this enzyme catalyzes pseudouridylation after splicing has been completed. This hypothesis receives further support from our finding that dyskerin also efficiently introduces pseudouridines into reporter mRNAs that lack introns. In cells lacking dyskerin, translation was enhanced globally by approximately 40%, indicating that mRNA pseudouridylation by the H/ACA complex attenuates translation. This effect required the catalytic activity of dyskerin, because reintroduction of wild-type, but not catalytically inactive dyskerin normalized protein production.

Several studies have demonstrated that the presence of pseudouridine in reporter mRNA can slow down its translation (*15*, *17*, *73*). For example, pseudouridine residues in mRNA can prevent proper positioning of tRNAs carrying amino acids in the ribosome, thereby reducing the rate of amino acid incorporation into the growing peptide chain (*14*). Pseudouridine residues can also trigger premature termination of translation, due to stalling and collision of ribosomes translating mRNA modified in this manner (*17*).

Although global translation was elevated following reduction of dyskerin-mediated pseudouridylation, we propose that the exact effect of pseudouridylation on translation depends on the extent and position of the pseudouridine residues within the mRNA. Therefore, it is possible or even likely that pseudouridylation by dyskerin enhances the translation of certain mRNAs. This idea is supported by our finding that in the absence of dyskerin, the association of one of our candidate mRNAs (*SRRM2*) with polysomes was not enhanced, even though it contained fewer pseudouridine residues.

Moreover, it is noteworthy in this context that incorporation of pseudouridine instead of uridine into synthetic mRNA prevents the activation of host defense RNA sensors, including RNase L and PKR, thereby enhancing RNA stability and translation (*12*, *19*, *74*). Although the realization that incorporation of pseudouridine can reduce immunogenicity and increase RNA stability has been pivotal in the development of mRNA therapy, it remains unclear whether endogenous incorporation has the same effects. Notably, in response to metabolic stress snoRNAs can bind and activate PKR (*75*).

While translation was enhanced following short-term depletion of dyskerin, long-term depletion attenuated translation. The latter effect involved defects in ribosome biogenesis and the established role of dyskerin in modification and processing of rRNA. Therefore, dyskerin appears to regulate protein synthesis at several levels, including suppression of translation by mRNA pseudouridylation and global enhancement through pseudouridylation of rRNA.

Here, we also found that in cells from patients with dyskeratosis congenita who carried mutations in *DKC1*, both mRNA and rRNA pseudouridylation are defective. Consistently, disease-associated mutations in dyskerin alter its binding to RNA or enhance the localization of complexes containing the mutant protein in splicing speckles, with the latter observation raising the possibility that mRNA pseudouridylation also occurs in these speckles. Although it remains unclear whether and in what manner defects in mRNA pseudouridylation may contribute to the clinical manifestations of dyskeratosis congenita, altered pseudouridylation of internal ribosome entry sites (IRES) could play a role, because translation of mRNAs containing these IRES elements was impaired in the same patients (*38*, *76*). This observation certainly warrants further investigation, and the light it sheds on the underlying causes of this fatal disorder can potentially lead to improved therapies.

## MATERIALS AND METHODS

### Cell culture and treatments

The cells and culture conditions used are shown in table S2. All cells were maintained at 37°C under 5% CO_2_ in humidified incubators. When indicated, cells were treated with 250 nM flavopiridol (Sigma-Aldrich), 1 μM triptolide (Sigma-Aldrich), or 100 nM pladienolide B (Santa Cruz Biotechnology) for 2 hours by adding the drug directly to the culture medium. To induce expression of the exogenous mRNA stably inserted into U2OS 2-6-3 cells, cells were treated with doxycycline (1 μg/ml; Sigma-Aldrich) for 3 hours.

Primary patient cell lines were derived from participants in the National Cancer Institute’s Institutional Review Board–approved longitudinal cohort study of inherited bone marrow failure syndromes (clinicaltrials.gov NCT00027274, https://marrowfailure.cancer.gov) (*36*). All study participants or their legal guardians provide informed consent in accordance with Health and Human Services regulation 45 CFR 46.

### Transfection

Cells were transfected with siRNAs (15 nM) using RNAiMAX (Thermo Fisher Scientific) and/or plasmids (1 μg) using Lipofectamine 2000 (Thermo Fisher Scientific) in accordance with the manufacturer’s instructions. The siRNAs and plasmids used are listed in table S3.

### Immunofluorescence

Cells were grown on coverslips, fixed with 4% paraformaldehyde for 10 min, and permeabilized with 0.1% Triton X-100 in phosphate-buffered saline (PBS) for 5 min at room temperature. The coverslips were then treated with blocking buffer (2% bovine serum albumin, 5% glycerol, 0.2% Tween 20, and 0.1% NaN_3_) for 1 hour at room temperature and incubated with primary antibody for 1 hour in blocking buffer at room temperature or overnight at 4°C. Coverslips were washed in PBS and incubated with secondary antibody and 4′,6-diamidino-2-phenylindole (0.1 μg/ml) in blocking buffer for 1 hour at room temperature, washed with PBS, and mounted with Mowiol 4-88 (Cold Spring Harbor protocols). All antibodies used are listed in table S4.

For pre-extraction before fixation, cells were incubated for 5 min at room temperature in cytoskeleton buffer [CSK; 10 mM Pipes (pH 7.0), 100 mM NaCl, 300 mM sucrose, 3 mM MgCl_2_, and 0.7% Triton X-100]. The permeabilization step was skipped, and cells were directly immersed in blocking buffer. For RNase A treatment, PureLink RNase A (Invitrogen) was added to the CSK buffer to a final concentration of 300 μg/ml, and cells were incubated for 5 min at room temperature.

### Microscopy and image analysis

Images were acquired with an LSM700 confocal microscope (Zeiss), mounted on an Axio Observer.Z1 (Zeiss) equipped with a Plan-Apochromat 63×/1.4 oil immersion lens. For images that were not to be analyzed, Z-stacks were acquired and processed with the Zen 2012 Black software (Zeiss) to produce maximum intensity projection images. For analysis of the intensity of signals in speckles, single-plane images were acquired on the focal plane that showed the highest intensity. Image analysis was performed in CellProfiler (v3.1.9) using a custom pipeline. Data analysis was performed using R (v4.1.1). Intensity profiles over speckles were generated using Fiji (v2.3.0).

### Chromatin immunoprecipitation

Six million to 10 million cells were cross-linked for 5 min at room temperature with 1% methanol-free formaldehyde (Thermo Fisher Scientific). After quenching with 125 mM glycine, cells were washed twice in ice-cold PBS and collected by scraping. Pellets were resuspended in buffer A [5 mM Pipes (pH 8.0), 85 mM KCl, and 0.5% NP-40 substitute] and incubated on ice for 10 min. After centrifugation (5000 rpm for 5 min at 4°C), the supernatants containing the cytoplasm were removed and the nuclei-containing pellets were washed in buffer A without detergent. Pellets were then resuspended in shearing buffer [10 mM tris-HCl (pH 8.0), 1 mM EDTA (pH 8.0), and 0.1% SDS] and sonicated with a ME220 focused-ultrasonicator (Covaris). After clarification by centrifugation (13,000 rpm for 10 min at 4°C), the supernatant was adjusted in composition to radioimmunoprecipitation assay (RIPA) buffer. Equal amounts of chromatin (as measured by DNA concentration) were incubated rotating overnight at 4°C with preconjugated beads (20 μl of Dynabeads and 3 μg of antibody) in RIPA buffer [10 mM tris-HCl (pH 8.0), 1 mM EDTA, 200 mM NaCl, 1% Triton X-100, 0.1% sodium deoxycholate, and 0.1% SDS]. Immunoprecipitates were washed once with each of the following: RIPA buffer, RIPA buffer high salt [10 mM tris-HCl (pH 8.0), 1 mM EDTA, 500 mM NaCl, 1% Triton X-100, 0.1% sodium deoxycholate, and 0.1% SDS], and LiCl buffer [10 mM tris-HCl (pH 8.0), 1 mM EDTA (pH 8.0), 250 mM LiCl, 0.5% NP-40 substitute, and 0.5% sodium deoxycholate], and twice with TE buffer [10 mM tris-HCl (pH 8.0) and 1 mM EDTA]. The beads were then resuspended in TE buffer with 0.5% SDS supplemented with proteinase K (New England Biolabs) and RNase A (Thermo Fisher Scientific) and incubated with shaking at 65°C for 4 hours. The DNA was recovered by phenol:chloroform:isoamyl alcohol extraction followed by ethanol precipitation using standard procedures and resuspended in TE buffer.

Sequencing libraries were generated using the NEBNext Ultra II DNA Library Prep Kit for Illumina (New England Biolabs) in accordance with the manufacturer’s instructions and sequenced single-end 50 bp with a HiSeq 2500 (Illumina) by Novogene (Hong Kong) or single-end 75 bp with a NextSeq 550 (Illumina) at the core facility for Bioinformatics and Expression Analysis at Karolinska Institutet (Huddinge, Sweden).

For quantitative reverse transcription PCR (RT-qPCR), enrichment of a particular DNA fragment was determined in a 96-well plate format with a 7500 Fast Real-Time PCR System (Thermo Fisher Scientific) using the fast SYBR green master mix (Thermo Fisher Scientific) and equal volumes of DNA. Enrichments in immunoprecipitated samples were calculated as percentage of input recovery. Primers are listed in table S3.

### RNA extraction, reverse transcription, and RT-qPCR

Total RNA was extracted with TRIzol reagent (Thermo Fisher Scientific) according to the manufacturer’s instructions, using 5PRIME Phase Lock Gel heavy tubes (Quantabio) to separate the aqueous phase from the interphase/organic phase. For cDNA generation, equal amounts of RNA were retrotranscribed with Superscript IV reverse transcriptase and random hexamer primers (all from Thermo Fisher Scientific) according to the manufacturer’s instructions. Enrichment of a particular RNA was determined by RT-qPCR in a 96-well plate format with a 7500 Fast Real-Time PCR System (Thermo Fisher Scientific) using the fast SYBR green master mix (Thermo Fisher Scientific) and calculated according to the ΔΔCt method or as percentage of input recovery.

### Western blotting

Cells were harvested in Western blot lysis buffer [100 mM tris-HCl (pH 8), 150 mM NaCl, 1% NP-40 substitute, and 1% protease inhibitor cocktail] for 30 min on ice. After brief sonication (5 cycles 30” ON/30” OFF, high output) with a standard Bioruptor (Diagenode), lysates were centrifuged at 13,000 rpm for 15 min at 4°C, and protein concentrations were determined using the Bradford assay (Bio-Rad). Proteins were resolved by SDS–polyacrylamide gel electrophoresis (SDS-PAGE) (Thermo Fisher Scientific) and transferred to nitrocellulose membranes (Thermo Fisher Scientific). Blots were incubated with primary antibodies (table S4) diluted in blocking solution (5% milk, 0.1% Tween 20 in PBS) overnight at 4°C followed by horseradish peroxidase–conjugated secondary antibodies (Cell Signaling). Blots were developed using SuperSignal West Femto maximum sensitivity substrate (Thermo Fisher Scientific) according to the manufacturer’s instructions. Densiometric analyses were performed with Fiji (v2.3.0). For the puromycin incorporation assay, cells were treated with puromycin (1 μg/ml; Invivogen) for 10 min, incubated with fresh medium for further 30 min, and then harvested.

### Immunoprecipitation from chromatin fractions

Cells were harvested in PBS and pelleted. Pellets were resuspended in buffer A [5 mM Pipes (pH 8.0), 85 mM KCl, and 0.5% NP-40 substitute] and incubated on ice for 10 min. After centrifugation (5000 rpm for 5 min at 4°C), the supernatants containing the cytoplasm were removed and the nuclei-containing pellets were washed in buffer A without detergent. Pellets were then resuspended in NP-40 buffer with 137 mM NaCl [50 mM tris-HCl (pH 7.5), 137 mM NaCl, and 1% Igepal], passed four times through a 25 G needle to disrupt nuclear membranes, and centrifuged (4000 rpm for 10 min at 4°C). The pellet (containing chromatin) was separated from the supernatant (that contains soluble nuclear proteins), resuspended in NP-40 buffer with 400 mM NaCl [50 mM tris-HCl (pH 7.5), 400 mM NaCl, and 1% Igepal], and subjected to sonication (5 cycles 30” ON/30” OFF, low output) with a standard Bioruptor. After centrifugation (13,000 rpm for 10 min at 4°C), the NaCl concentration was adjusted with NP-40 buffer without NaCl [50 mM tris-HCl (pH 7.5) and 1% Igepal], and protein concentration was measured. Protein lysate per immunoprecipitation (50 μg) was incubated while rotating overnight at 4°C with preconjugated beads (5 μl of Dynabeads and 1 μg of antibody) in NP40 buffer 137 mM NaCl. After one wash in NP-40 buffer 137 mM NaCl and two washes in NP-40 buffer with 200 mM NaCl [50 mM tris-HCl (pH 7.5), 200 mM NaCl, and 1% Igepal], samples were resolved by SDS-PAGE following the Western blotting protocol.

For RNase A treatment, after the last wash, beads were resuspended in NP-40 buffer 200 mM NaCl with or without RNase A (100 ng/ml; Thermo Fisher Scientific), and incubated on a thermomixer with gentle shaking for 30 min at 37°C. After another two washes in NP-40 buffer 200 mM NaCl, samples were resolved by SDS-PAGE following the Western blotting protocol.

RIP from chromatin fractions was performed like protein coimmunoprecipitation, except that, after the final washes, the RNA was purified and analyzed following the procedure described above. For the RIP experiment including a spike-in *GFP* mRNA, 50 ng of i–transcribed *GFP* mRNA with 100% pseudouridine content were added per 100 μg of protein lysates, followed by aliquoting the lysate into input and the IP mixtures.

### RIP from purified RNA

Total RNA from 6 million to 10 million cells was obtained as described above. From this, mRNA was isolated using Dynabeads Oligo(dT)_25_ (Thermo Fisher Scientific) according to the manufacturer’s instructions. The purified mRNA was then fragmented with RNA fragmentation reagents (Thermo Fisher Scientific) for 1 min at 70°C and precipitated with ethanol.

After precipitation, 250 to 500 ng of fragmented mRNA were incubated overnight at 4°C with preconjugated beads (5 μl of Dynabeads and 1 μg of antibody) in IPP buffer [10 mM tris-HCl (pH 7.5), 150 mM NaCl, and 0.1% Igepal]. Immunoprecipitates were washed twice with each of the following: IPP buffer, IPP buffer low salt [10 mM tris-HCl (pH 7.5), 50 mM NaCl, and 0.1% (v/v) Igepal], and IPP buffer high salt [10 mM tris-HCl (pH 7.5) and 500 mM NaCl]. Beads were eluted directly in TRIzol reagent (Thermo Fisher Scientific) and the RNA recovered and quantified as above.

### RNA sequencing

Cells were harvested in PBS and pelleted. Pellets were resuspended in buffer A [5 mM Pipes (pH 8.0), 85 mM KCl, and 0.5% NP-40 substitute] and incubated on ice for 10 min. After centrifugation (5000 rpm for 5 min at 4°C), the supernatants containing the cytoplasm were removed and the nuclei-containing pellets were washed in buffer A without detergent. Nuclear RNA was extracted with TRIzol reagent (Thermo Fisher Scientific) according to the manufacturer’s instructions, using 5PRIME Phase Lock Gel heavy tubes (Quantabio) to separate the aqueous phase from the interphase/organic phase.

Sequencing libraries were generated using the RiboCop rRNA depletion kit followed by the CORALL Total RNA-Seq library preparation kit (both from Lexogen), following the manufacturer’s instructions and thereafter sequenced paired-end 50 bp with a NovaSeq 6000 (Illumina) at the Science for Life Laboratory National Genomics Infrastructure (Stockholm, Sweden).

### Individual-nucleotide resolution cross-linking and immunoprecipitation

U2OS cells were grown in 15 cm dishes, and protein-RNA complexes were cross-linked with 1500 J/m^2^ 254 nm UV light. After harvesting, cells were resuspended in cytoplasmic lysis buffer [50 mM tris-HCl, (pH 7.4), 10 mM NaCl, 0.5% Igepal, 0.25% Triton, and 1 mM EDTA], rotated at 4°C for 5 min, spun down, and washed. Nuclei were then resuspended in lysis buffer and subjected to the protocol described in (*77*). Briefly, nuclear lysates were treated with 0.4 U RNase I (Thermo Fisher Scientific) and 4 U TurboDNase (Thermo Fisher Scientific) per 1 ml of cell lysate at 1 mg/ml protein concentration. Beads coupled with mouse immunoglobulin G, dyskerin, or GAR1 antibodies were used to precipitate protein-RNA complexes, after which RNA was ligated to a pre-adenylated infra-red labeled IRL3 adaptor. Complexes were resolved through SDS-PAGE and cut from the nitrocellulose membrane. RNA was then released from the membrane with proteinase K digestion, recovered by precipitation, and retrotranscribed with Superscript IV Reverse Transcriptase (Thermo Fisher Scientific). The resulting cDNA was purified using AMPure XP beads (Beckman-Coulter), then circularized using CircLigase II (Epicentre), and purified again with AMPure XP beads. After PCR amplification, libraries were size-selected with Ampure XP beads and quality-controlled for sequencing. Libraries were sequenced single-end 100 bp with a HiSeq 4000 (Illumina) by the sequencing facility at the Francis Crick Institute (London, UK).

### Liquid chromatography–tandem mass spectrometry

Total RNA was isolated from chromatin-fractions of siControl or siDyskerin-treated U2OS cells (48 hours of knockdown) using TRIzol. The aqueous phase was then mixed with 1 volume of 70% EtOH and passed through a RNeasy Mini Spin Column as per the Qiagen RNeasy kit protocol, to remove RNAs below 200 nt (including tRNA). On-column DNA digestion (RNase-Free DNase Set, Qiagen) was performed to remove potential gDNA contaminant. After elution from the column, mRNA was extracted as described above but with three sequential poly (dT) extractions, to increase purity and remove potential contaminating ncRNA. RNA was enzymatically digested using benzonase (Santa Cruz Biotechnology) and nuclease P1 (Sigma-Aldrich) in 10 mM ammonium acetate (pH 6.0) and 1 mM MgCl_2_ at 40°C for 1 hour, added ammonium bicarbonate to 50 mM phosphodiesterase I and alkaline phosphatase (Sigma-Aldrich), and incubated further at 37°C for 1 hour. Digested samples were precipitated with 3 volumes of acetonitrile, and supernatants were lyophilized and dissolved in a solution of stable isotope-labeled internal standards (I.S.; see mass list) for LC-MS/MS analysis. An Agilent 1290 Infinity II UHPLC system with an ZORBAX RRHD Eclipse Plus C18 150 by 2.1 mm (1.8 μm) column protected with an ZORBAX RRHD Eclipse Plus C18 5 by 2.1 mm (1.8 μm) guard (Agilent) was used for chromatographic separation. The mobile phase consisted of A: water and B: methanol (both added 0.1% formic acid) at 0.22 ml/min, for modifications starting with 5% B for 0.5 min followed by 2.5 min of 5 to 20% B, 3.5 min of 20 to 95% B, and 4 min re-equilibration with 5% B. Canonical nucleosides were chromatographed with a 4 min gradient of 5 to 95% B and 4 min re-equilibration with 5% B. Mass spectrometric detection was performed using an Agilent 6495 Triple Quadrupole system monitoring the mass transitions 243.1/153.1 (pseudouridine, Ψ) in negative mode, and 261.1/145.1 [d_3_-5hm(dC), coelutes with Ψ, I.S. for Ψ], 268.1/136.1 (A), 284.1-152.1 (G), 244.1/112.1 (C), 245.1/113.1 (U), 282.1/150.1 (m^6^A), 298.1/166.1 (m^7^G), 285.1/153.1 (d_3_-m6A I.S.), 273.1/136.1 (^13^C_5_-A I.S.), and 246.1/114.1 (d_2_-C I.S.) in positive mode.

### Polysome fractionation

A total of 1.8 million MCF7 cells were plated in a 15 cm dish and the following day transfected for 48 hours with control siRNA or an siRNA targeting dyskerin. After harvesting, cells were subjected to polysome fractionation as described previously (*78*). Fractions were collected as described in (*78*), and equal volumes from fractions corresponding to more than three ribosomes were pooled and the RNA extracted. Equal amounts of RNA were used for reverse transcription and qPCR, as described above. Notably, we used MCF7 because these cells have larger polysomes than U2OS cells. 

### In vitro transcription, m^7^G capping, and poly(A) tailing

For templates, PCR fragments containing the T7 promoter and the *GFP* coding sequence were amplified from a pCMV-T7-GFP plasmid (Addgene) using the Advantage HD Polymerase Mix (Takara) following the manufacturer’s instructions. The PCR product (200 ng) was in vitro transcribed using the Megascript T7 transcription kit (Thermo Fisher Scientific) following the manufacturer’s instructions, with the indicated percentages of Pseudo-UTP (Jena Bioscience) used in place of the uridine triphosphate provided with the kit as the only variation. RNA was recovered via LiCl precipitation.

In vitro–transcribed mRNA (60 μg) was then capped and poly-A tailed with the ScriptCap m^7^G Capping system (CellScript) and the A-Plus Poly(A) Polymerase Tailing Kit (CellScript), according to the manufacturer’s instructions. The processed RNA was recovered via TRIzol extraction and used for subsequent experiments.

### In vitro translation

A total of 0.5 to 2 μg of purified RNA were translated using wheat germ extract (Promega) at 25°C for 90 min in a final reaction volume of 12.5 μl according to the manufacturer’s instructions. Protein output was analyzed via Western blotting as described above.

### ChIP-seq data processing

Sequencing quality checks were performed on all experiments using FastQC (v0.11.9). Adapter sequences were removed using TrimGalore (v0.6.4_dev), and reads shorter than 20 nt were discarded. Reads were aligned to the human reference genome (GRCh38/hg38) using bwa-mem (v0.7.17-r1188) with default options. Reads that failed to align and those with MAPQ ≤30 were filtered out, and PCR duplicates were discarded using the Picard MarkDuplicates (v2.18.11) module. Genome coverage tracks in bigWig format were generated using the bamCoverage module from deeptools (v3.2.1) with -*-binSize 50 --normalizeUsing CPM* options. Moreover, coverage tracks were normalized against their background (i.e., Input) using the bigwigCompare module from deeptools, with *--skipZeroOverZero --skipNonCoveredRegions* options, and replicates were merged with the same procedure but adding the *--operation mean* option.

### Metadata genomic profiles

The metadata profiles were generated using the normalized coverage bigWig files and the computeMatrix module from deeptools. The output matrices were then imported into R (v4.1.1) for plotting. Depending on the specific analysis, the submodules reference-point or scale-regions were used respectively to summarize the signal flanking features of interest (*−-upstream 5000 --downstream 5000 --missingDataAsZero --skipZeros*), such as TSS and TES, and across regions of variable length (*−-regionBodyLength 2000 --upstream 2000 --downstream 2000 --missingDataAsZero --skipZeros*), such as whole genes.

### RNA-seq data processing

Sequencing quality checks were performed on all experiments using FastQC (v0.11.9). Adaptor sequences were removed using TrimGalore (v0.6.4_dev) with default parameters. Reads were filtered against human rRNA and tRNA sequences obtained from the NCBI using Bowtie2 (v2.4.1) with the option *--sensitive-local*. Reads that failed to align were used as input for STAR (v2.7.0e) and mapped to the human reference genome (GRCh38/hg38) using GENCODE (v27) gene annotation as reference, with the following parameters: *--twopassMode Basic --alignSJoverhangMin 8 --alignSJDBoverhangMin 1 --sjdbScore 1 --outFilterMultimapNmax 1 --outFilterType BySJout --outFilterMismatchNmax 999 --outFilterMismatchNoverReadLmax 0.04 --outSAMattributes All --outSAMtype BAM SortedByCoordinate --alignIntronMin 20 --alignIntronMax 1000000 --alignMatesGapMax 1000000*. PCR duplicates were removed using Picard MarkDuplicates (v2.18.11) with default parameters. Last, genome coverage tracks in bigWig format were generated using the deduplicated unique alignments and the bamCoverage module from deeptools (v3.2.1) with *--normalizeUsing CPM --binSize 20 --smoothLength 60* options, and *--filterRNAstrandZ* for the selection of reads mapping to the forward or reverse strands.

### Transcript quantification

Transcript expression quantification was carried out using Salmon (v1.2.0) in pseudo-alignment mode, with library type (*−l ISR*) and correction of sequence-specific (*−-seqBias*) and position-specific (*−-posBias*) biases, and the GENCODE (v27) annotation and corresponding transcriptome as references. Transcripts per million (TPM) values obtained with Salmon were imported into *R* using the tximport package with ‘lengthScaledTPM’ scaling method and no scaling for differential gene expression analysis. Counts were summarized at gene level by means of the tximport() function. Gene expression levels normalization across samples and differential gene expression calculations were performed using the DESeq2 R package, and the IHW R package for Independent Hypothesis Weighting. The significance *P* values were adjusted for multiple hypotheses testing by the Benjamini-Hochberg method, and a false discovery rate (FDR) threshold of 0.01 was used to extract genes significantly changing their expression between conditions. Differential alternative splicing events were detected using rMATS (v4.1.2). Events with an FDR ≤ 0.01 were considered to be statistically significant.

### iCLIP-seq data processing

Reads were processed according to standardized iCLIP analysis methods using the iMaps webserver. Briefly, sequencing quality checks were performed on all experiments using FastQC (v0.11.9). Adapter sequences were removed using Cutadapt and the following parameters: *-q 20 -m 10 --times 1 --error-rate 0.1 -O 3*. Reads were mapped to the human reference genome (GRCh38/hg38) using GENCODE (v27) gene annotation as reference and STAR (v2.7.0e), with the following parameters: *--outSAMtype BAM SortedByCoordinate --outReadsUnmapped Fastx --outFilterType Normal --outFilterMismatchNmax 2 --alignEndsType EndToEnd --outSAMunmapped Within --outSAMattributes Standard*. Mapped reads were analyzed with the iCount (v2.0.1.dev) software (https://github.com/tomazc/iCount): the xlsites module (−*-group_by start --quant cDNA --mismatches 1 --mapq_th 0 --multimax 50 --gap_th 1000000 --ratio_th 0.001*) was used to extract the cross-link sites; the bedgraph module was used to generate genomic coverage tracks; the peaks module (*−-group_by gene_id --half_window 3 --fdr 0.05 --perms 100 --rnd_seed 42*) was used to identify the regions significantly enriched in protein binding events.

### Statistics and reproducibility

Data presented as bar graphs were analyzed using Prism GraphPad (v9.2.0), and the test performed to compare the different groups is indicated in the figure legends. The statistical significance of pseudouridine reduction following dyskerin knockdown was tested through a one-tailed test because we expected a decrease, due to the fact that loss of dyskerin was previously shown to result in diminished RNA pseudouridylation (*6*, *62*, *64*). Data presented as boxplots were analyzed with R (v4.1.1), and the different groups were compared using the Wilcoxon rank sum test. *P* value notations are provided as follows: *P < 0.05, **P < 0.01, ***P < 0.001, and ****P ≤ 0.0001. A combination of biological replicates and independent experiments was used to ensure reproducibility of results.

## Supplementary Materials

### This PDF file includes:

Figs. S1 to S7 Tables S1 to S5 References
