# Supplementary material for: Control of protein synthesis through mRNA pseudouridylation by dyskerin
Source: Sci Adv. 2023 Jul 28;9(30):eadg1805. doi: 10.1126/sciadv.adg1805 (PMC10381945; doi:10.1126/sciadv.adg1805)
Supplement: Supplementary file 1 — Figs. S1 to S7 Tables S1 to S5 References [file sciadv.adg1805_sm.pdf]

Science Advances
AAAS

Supplementary Materials for

Control of protein synthesis through mRNA pseudouridylation by dyskerin

Chiara Pederiva *et al.*

Corresponding author: Marianne Farnebo, marianne.farnebo@ki.se; Chiara Pederiva, chiara.pederiva@ki.se

Sci. Adv. **9**, eadg1805 (2023)
DOI: 10.1126/sciadv.adg1805

This PDF file includes:

Figs. S1 to S7
Tables S1 to S5
References

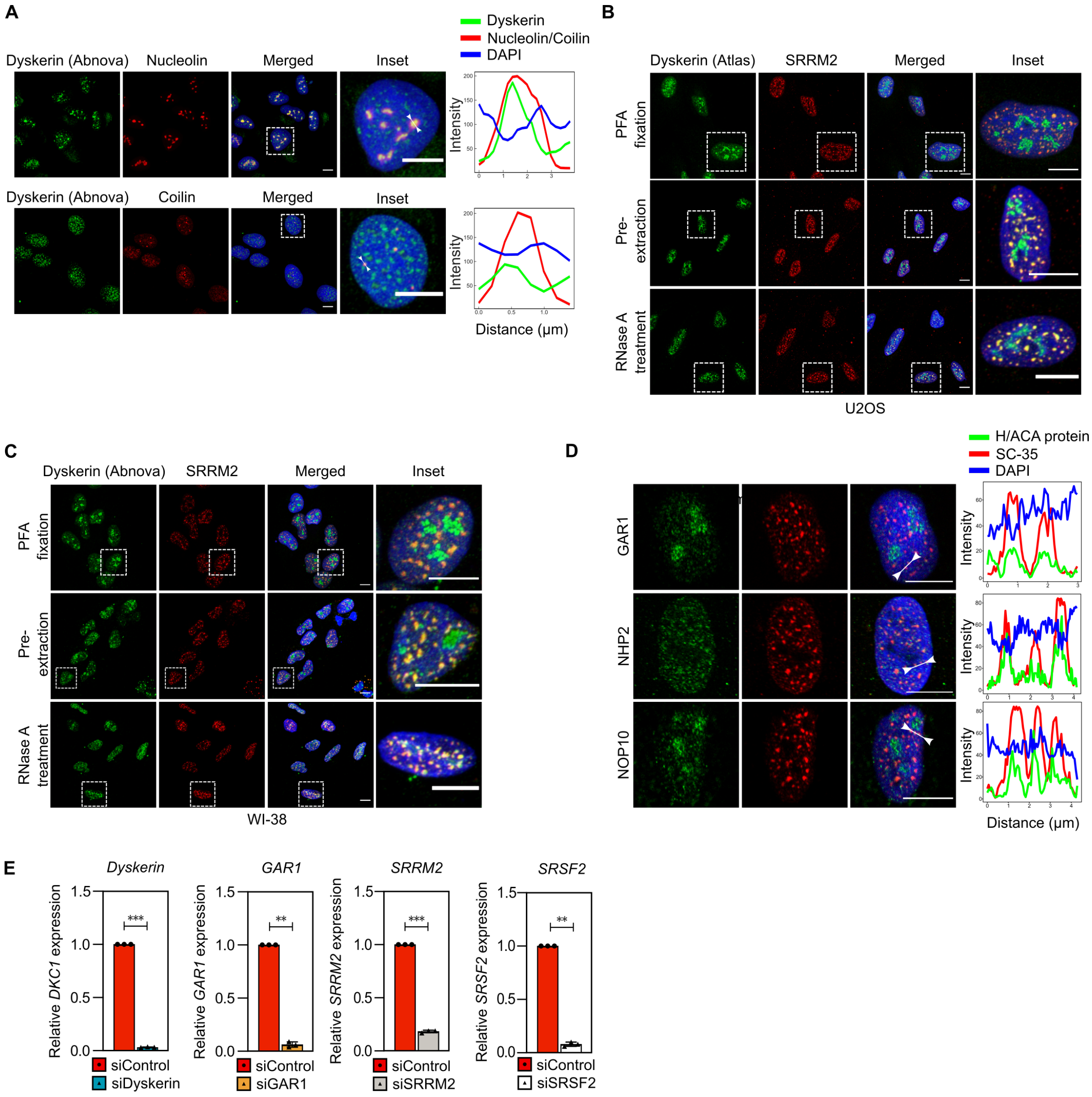

Fig. S1. Detection of dyskerin in nuclear speckles employing different antibodies and cell types

(A) U2OS cells immunostained for dyskerin (green, Abnova antibody) and nucleolin (marker of nucleoli; red) or coilin (marker of Cajal bodies; red). Intensity profiles were calculated across the nucleolus and Cajal body indicated between the two arrows. Scale bar (white line) = 10µm.

(B) U2OS cells immunostained for dyskerin (green, Atlas antibody) and the speckle protein SRRM2 (red). PFA fixation indicates fixation with 4% formaldehyde followed by permeabilization; 'Pre-extraction' indicates permeabilization of cells with cytoskeleton buffer 5 minutes prior to fixation with PFA; 'RNase A' is the same as 'pre-extraction' but with addition of 300 µg/ml RNase A to the cytoskeleton buffer. The dyskerin antibody used here is a second independent antibody raised in another species compared to the one used in Figure 1A. Scale bar = 10µm.

(C) WI-38 fibroblasts pre-extracted as above and immunostained for dyskerin (green, Abnova antibody) and SRMM2 (red). Scale bar = 10µm.

(D) U2OS cells were treated with RNase A and immunostained using antibodies for GAR1, NHP2 or NOP1 (green) and the speckle marker SC-35 (red). Intensity profiles were calculated above the speckle(s) indicated between the two arrows. Scale bar = 10µm.
(E) U2OS cells were subjected to siRNA treatment for 48 hours and then analyzed by qPCR for expression of the mRNA indicated. RNA levels were normalized to those of beta-actin mRNA and are shown relative to the siControl sample (mean ± SD, n=3 independent experiments). **** $p \leq 0.0001$, as determined by a paired ratio two-tailed *t*-test.

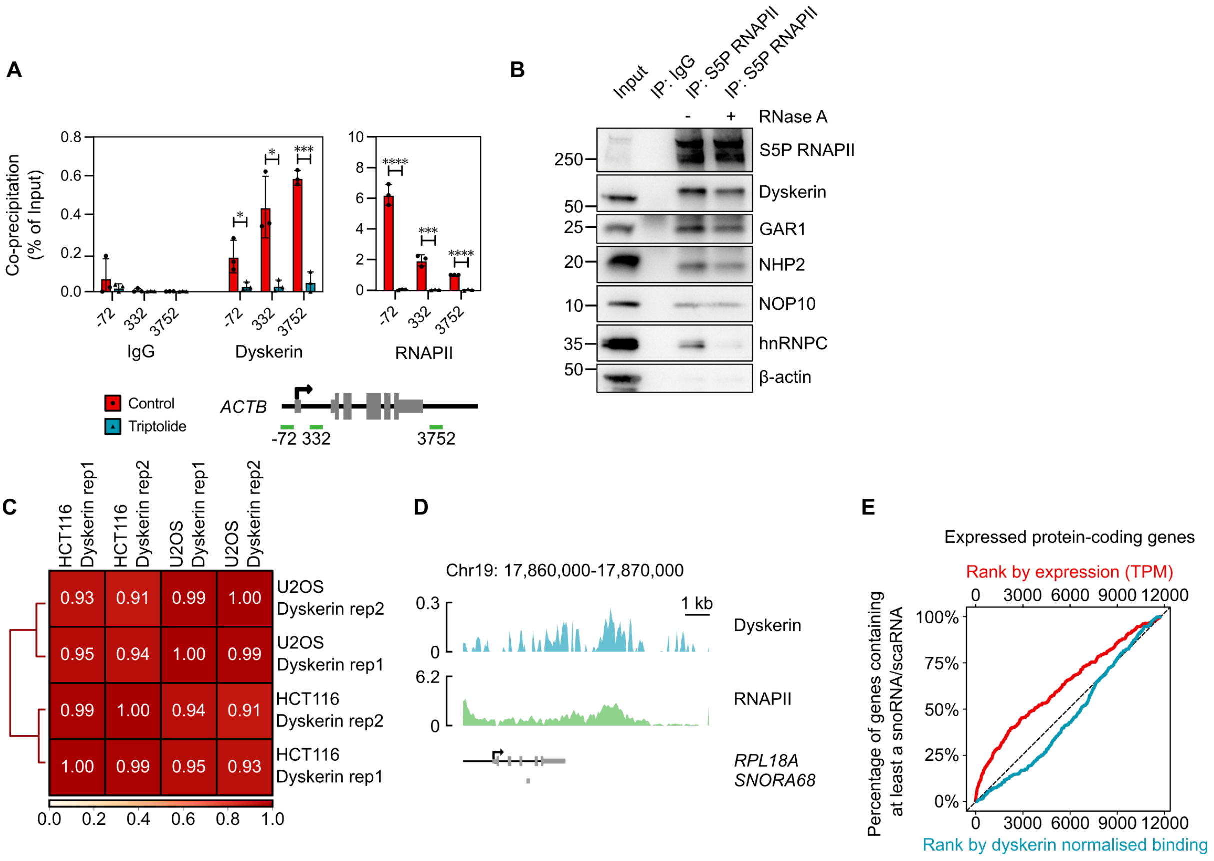

Fig. S2. Dyskerin binds to genes through RNAPII in different cell lines

(A) ChIP-qPCR results showing dyskerin and RNAPII (precipitated with the 8WG16 antibody) enrichment at the *ACTB* locus in U2OS cells treated or not with triptolide for 2 hours. The PCR-amplified regions inside the *ACTB* gene are indicated with green lines in the scheme. Data are represented as mean ± SD, n=3 independent experiments. * $p<0.05$, *** $p<0.001$, **** $p<0.0001$, as determined by unpaired, two-tailed Student's *t*-test.

(B) Immunoprecipitation of RNAPII or IgG from the chromatin fraction of U2OS cells with or without RNase A treatment followed by western blotting of the proteins indicated. A characteristic blot is shown.

(C) Heatmap showing the correlations between dyskerin ChIP-seq binding profiles in U2OS and HCT116 cells. The numbers shown represent Spearman's correlation coefficient.

(D) Shown is an example of the ChIP-seq profile of dyskerin and RNAPII at the *RPL18A* gene (*i.e.*, the host gene for *SNORA68*) in U2OS cells. The ChIP-seq signal is normalized to input, and averaged across of 2 independent experiments.

(E) Cumulative distribution of genes that contain at least one snoRNA or scaRNA inside their introns across genes ranked according to the normalized dyskerin binding in ChIP-seq (blue) or RNA-seq expression (TPM; red). Dyskerin ChIP binding and RNA expression were ranked from highest to lowest from left to right.

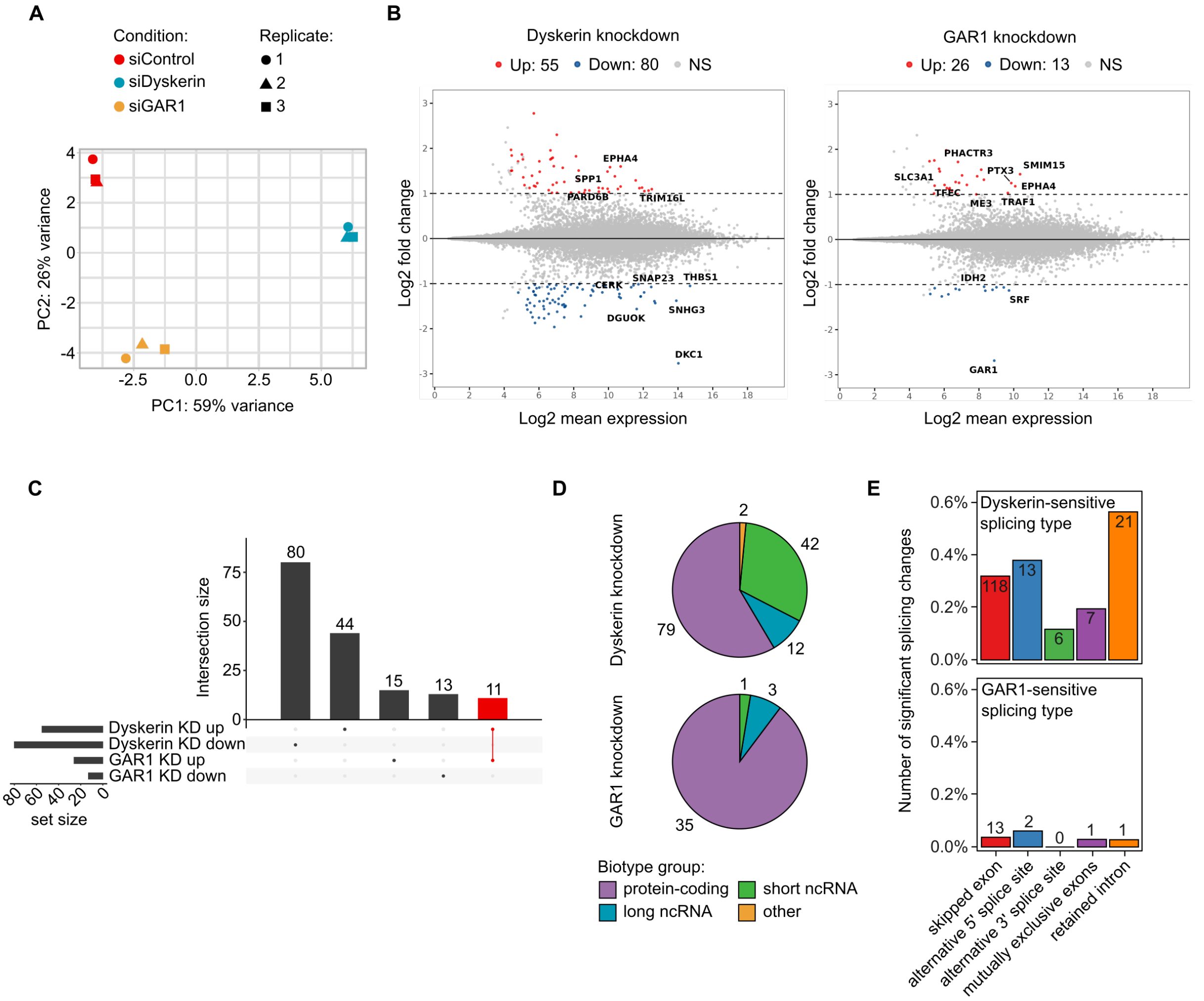

Fig. S3. Knockdown of dyskerin or GAR1 causes no major change in the steady-state level of RNA

(A) PCA plot showing the dispersion of RNA-seq samples obtained from nuclear fractions of U2OS cells treated with the indicated siRNA for 48 hours (3 biological replicates).

(B) MA plot of the differentially expressed genes after dyskerin and GAR1 knockdown (as determined by DESeq2) in the RNA-seq samples described above. Genes were considered differentially expressed if their absolute $\log_2$ fold change over control was higher than 1.

(C) Graph showing the number of transcripts deregulated by dyskerin or GAR1 knockdown (KD) in the RNA-seq samples described above. The dots indicate the sets to which the bars refer to, while the lines indicate the intersections between the sets.

(D) Pie chart of RNA differentially expressed after dyskerin or GAR1 knockdown in the RNA-seq samples described above, organized by RNA biotype group.

(E) Percentage of significant splicing changes in cells after dyskerin (top) or GAR1 (bottom) knockdown in the RNA-seq samples described above compared to control cells, divided by the type of alternative splicing. The numbers indicate the number of different splicing events.

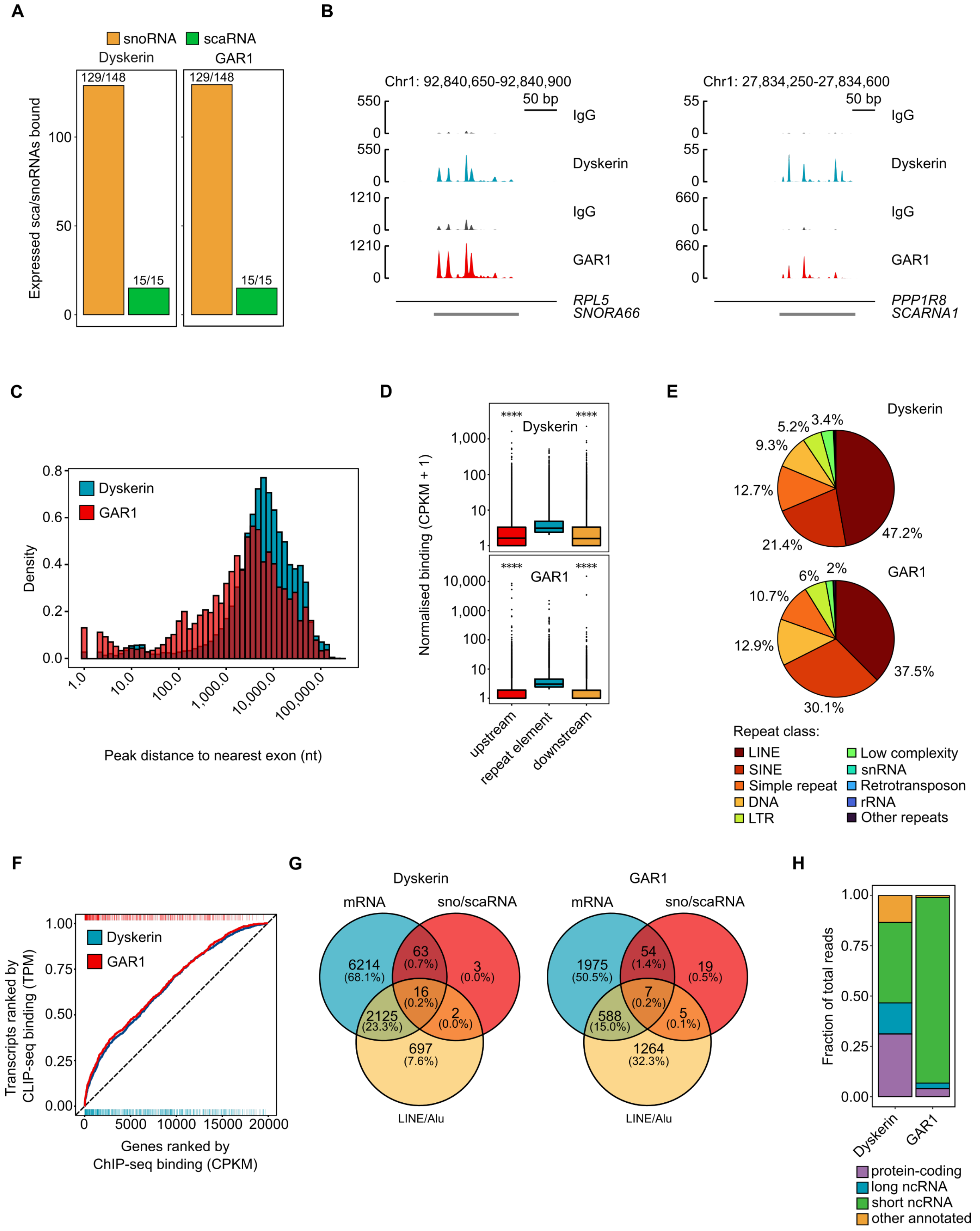

Fig. S4. Dyskerin and GAR1 bind to known non-coding RNAs, mRNAs and intronic repeats

(A) Fraction of the expressed sno/scaRNAs bound by dyskerin (left) or GAR1 (right) in U2OS cells. The numbers indicate the proportion of bound sno/scaRNAs over all the expressed ones**.**

(B) Shown are examples of dyskerin and GAR1 binding over *SNORA66* (top) and *SCARNA1* (bottom). The iCLIP signal is normalized to input, and averaged across 2 independent experiments.
(C) Distribution of the distance between dyskerin and GAR1 iCLIP peaks within introns, and the nearest exon boundary.
(D) Distribution of dyskerin and GAR1 iCLIP peaks inside intronic repeat elements and their sized-matched upstream and downstream regions. **** $p \leq 0.0001$, Wilcoxon rank-sum test. The graph shows that binding of dyskerin and GAR1 to repeat elements is specific, and not due to generic association of the two proteins with intronic sequences.
(E) Distribution of dyskerin and GAR1 iCLIP peaks among different classes of repeat elements.
(F) Cumulative distribution functions of protein-coding RNAs bound by dyskerin (blue) and GAR1 (red) across protein-coding genes ranked by dyskerin ChIP-seq binding. Dyskerin ChIP-seq binding was ranked from highest to lowest from left to right.
(G) Venn diagram showing distribution of binding of dyskerin or GAR1 to protein-coding RNAs (blue circles), sno/scaRNAs (red circles) and LINE/Alu RNA (yellow circles). The intersections represent sno/scaRNAs or LINE/Alu that are encoded from introns of expressed protein-coding mRNA bound by dyskerin or GAR1.
(H) Distribution of iCLIP reads from dyskerin and GAR1, divided by RNA biotype group, and normalized by their length and expression. Shown is the average of two independent experiments.

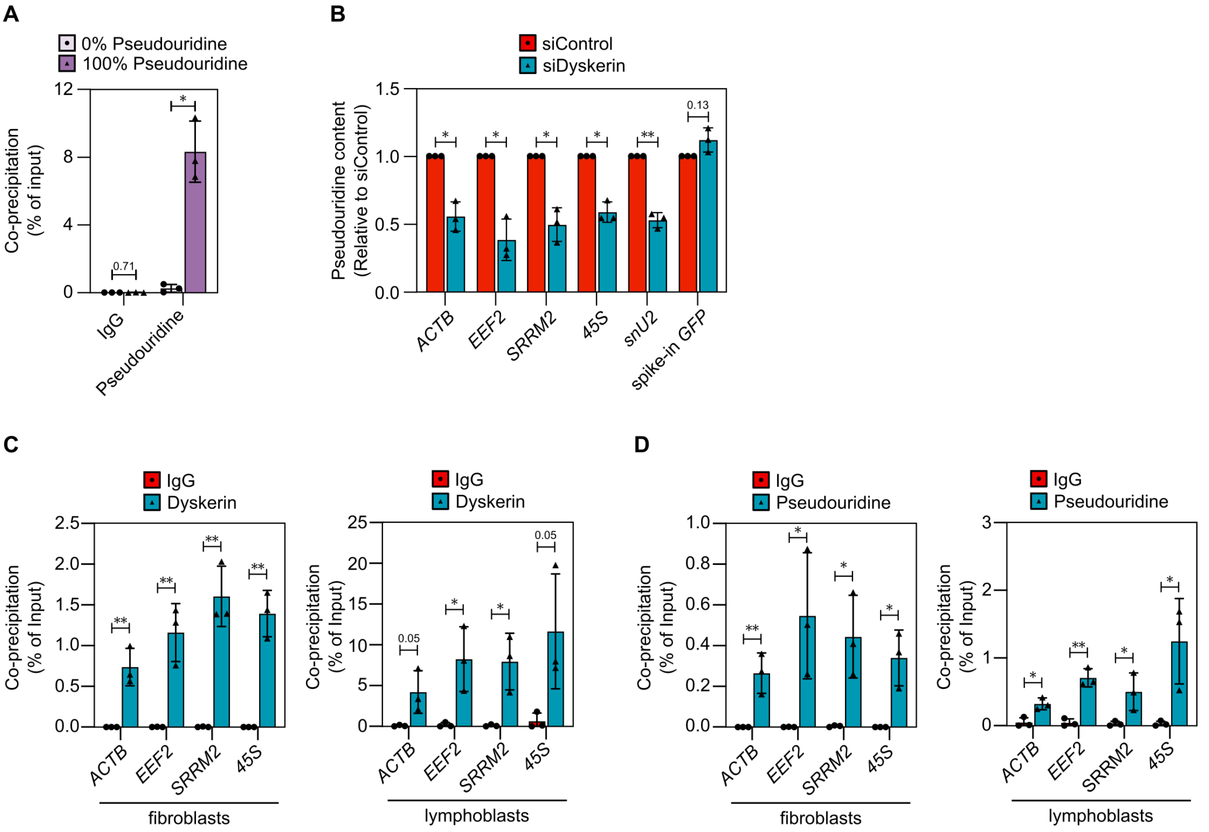

Fig. S5. mRNAs bind dyskerin and are pseudouridylated in fibroblasts and lymphoblasts

(A) *GFP* mRNAs containing only uridine (0% pseudouridine) or pseudouridine (100%) were subjected to RIP with an antibody against pseudouridine or IgG to verify its specificity. The graph shows the amount of co-precipitated RNA as percentage of input (mean ± SD, n=3) measured by qPCR. * $p<0.05$, ns non-significant, as determined by unpaired, two-tailed Student's *t*-test.

(B) Chromatin fractions of U2OS cells treated with the siRNA indicated for 48 hours were subjected to RNA immunoprecipitation using an antibody targeting pseudouridine. 50 ng of *in vitro*-transcribed *GFP* mRNA with 100% pseudouridine content were added per 100 µg of protein lysates prior to addition of antibody. The graph shows the amount of co-precipitated RNA (mean ± SD, n=3 independent experiments) measured by qPCR and presented as percentage of input relative to siControl. *** $p<0.001$, **** $p \leq 0.0001$, as determined by two-tailed paired ratio *t*-test.

(C) Chromatin fractions from primary fibroblasts (left) and immortalized lymphoblasts (right) from healthy donors were subjected to RNA immunoprecipitation using an antibody targeting dyskerin or a negative IgG control. The graph shows the amount of co-precipitated RNA as percentage of input (mean ± SD, n=3 independent experiments) measured by qPCR. * $p<0.05$, ** $p<0.01$, ns non-significant, as determined by two-tailed Student's *t*-test.

(D) Chromatin fractions from primary fibroblasts (left) and immortalized lymphoblast (right) from healthy donors were subjected to RNA immunoprecipitation using an antibody targeting pseudouridine or a negative IgG control. The graph shows the amount of co-precipitated RNA as percentage of input (mean ± SD, n=3 independent experiments) measured by qPCR. * $p<0.05$, ** $p<0.01$, as determined by two-tailed Student's *t*-test.

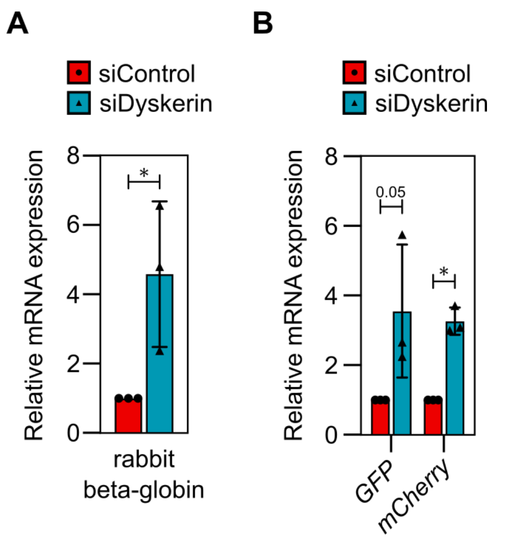

Fig. S6. Elevated expression of exogenous mRNAs in dyskerin-depleted cells

(A) U2OS 2-6-3 CLTon cells were treated for 48 hours with siRNA and for the last 3 hours with doxycycline to induce the expression of the exogenous rabbit beta-globin cassette and then analyzed by qPCR for expression of rabbit beta-globin. The RNA levels were normalized to those of beta-actin mRNA and are shown relative to the control sample (mean ± SD, n=3 independent experiments). * $p<0.05$, as determined by two-tailed paired-ratio *t*-test.

(B) U2OS cells were transfected with siRNA for 48 hours and for the last 6 hours with a plasmid encoding *GFP* or *mCherry* and then analyzed by qPCR for expression of *GFP* or *mCherry*. The RNA levels were normalized to those of beta-actin mRNA and are shown relative to the control sample (mean ± SD, n=3 independent experiments). * $p<0.05$, ** $p<0.01$, as determined by two-tailed paired ratio *t*-test.

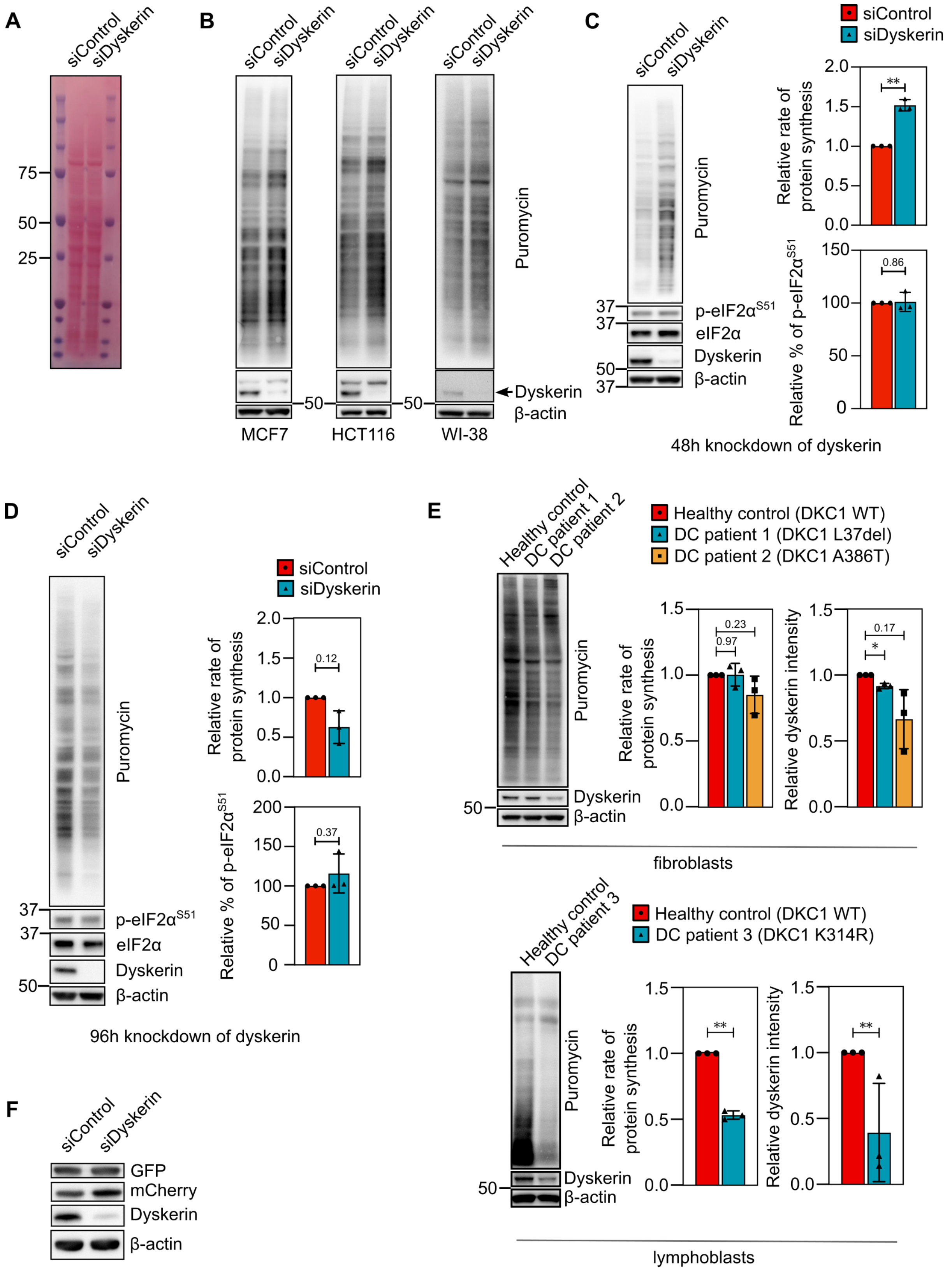

Fig. S7. Transient loss of dyskerin triggers an enhancement of protein synthesis, while prolonged dyskerin deficiency leads to aberrant rRNA processing and reduced translation

(A) Ponceau staining of a western blot membrane with lysates from U2OS cells treated with siRNA for 48 hours.
(B) MCF7, HCT116 and WI-38 cells were subjected to siRNA treatment for 48 hours, pulsed with puromycin for 10 minutes followed by 30 minutes recovery and western blotting. Shown are representative blots.
(C) U2OS cells were transfected with siRNA for 48 hours, pulsed with puromycin for 10 minutes followed by 30 minutes recovery, and extracted for western blotting. Shown are a representative blot and the densitometric quantification of puromycin (normalized to β-actin) and p-eIF2α^{S51} (normalized to β-actin and total eIF2α) levels relative to the levels of siControl (mean ± SD, n=3 biological replicates). ** $p<0.01$, as determined by two-tailed paired ratio *t*-test. The puromycin blot is the same as the one shown in Figure 6E.
(D) U2OS cells were transfected with siRNA for 96 hours, pulsed with puromycin for 10 minutes followed by 30 minutes recovery and extracted for western blotting. Shown are a representative blot and the densitometric quantification of puromycin (normalized to β-actin) and p-eIF2α^{S51} (normalized to β-actin and total eIF2α) levels relative to the levels of siControl (mean ± SD, n=3 biological replicates). The puromycin blot is the same as the one shown in Figure 6E. p values as determined by two-tailed paired ratio *t*-test.
(E) Fibroblasts (top) and lymphoblasts (bottom) from patients with dyskeratosis congenita (DC) and healthy donors were pulsed with puromycin for 10 minutes followed by 30 minutes recovery and extracted for western blotting. Shown are a representative blot and the densitometric quantification of puromycin and dyskerin normalized to β-actin and relative to the levels of healthy control (mean ± SD, n=3 independent experiments). * $p<0.05$, ** $p<0.01$, as determined by two-tailed paired ratio *t*-test.
(F) U2OS cells were transfected with siRNA for 48 hours and for the last 16 hours with a plasmid encoding GFP or mCherry followed by western blotting for the proteins indicated.

Table S1. LC-MS/MS

RNA type	Treatment	ψ/10^4 canonical rNs	m7G/10^4 canonical rNs	m6A/10^4 canonical rNs	m2,2G/10^4 canonical rNs	m6,6A/10^4 canonical rNs
		Modification per 10 000 canonical/unmodified ribonucleotides (rNs)				
mRNA	siControl 1	0,58388	1,63504	5,03868	0,13171	0,00151
mRNA	siDyskerin 1	0,45781	1,69066	4,97309	0,10229	0,00124
mRNA	siControl 2	1,80465	1,48353	6,76193	0,00379	0,01266
mRNA	siDyskerin 2	1,32319	1,52754	6,80145	0,01858	0,01105
mRNA	siControl 3	1,47012	1,24948	6,59909	0,01889	0,01560
mRNA	siDyskerin 3	1,23028	1,62373	7,27172	0,00579	0,01526
mRNA	siControl 4	0,85463	0,82978	5,36733	0,04564	0,03712
mRNA	siDyskerin 4	0,64966	1,15938	6,37077	0,00217	0,01528
Total RNA (mRNA depleted)	siControl 1	82,04668	1,11427	2,63879	0,57439	1,72413
Total RNA (mRNA depleted)	siDyskerin 1	56,10665	1,05374	2,12262	0,71871	1,57222
Total RNA (mRNA depleted)	siControl 2	54,98914	1,20096	1,73084	0,00583	1,55645
Total RNA (mRNA depleted)	siDyskerin 2	48,11022	1,35966	1,81726	0,01505	1,80174
Total RNA (mRNA depleted)	siControl 3	60,47893	1,23923	1,86190	0,00609	1,84182
Total RNA (mRNA depleted)	siDyskerin 3	54,52861	1,51189	1,99940	0,00624	2,02162
Total RNA (mRNA depleted)	siControl 4	61,42222	0,63452	1,16859	0,00962	1,00302
Total RNA (mRNA depleted)	siDyskerin 4	61,18129	1,08429	1,52026	0,00841	1,76056
					tRNA specific	18S rRNA specific

RNA type	Treatment	ψ/U (%)	m7G/G (%)	m6A/A (%)	m2,2G/G (%)	m6,6A/A (%)
		Modification relative to the unmodified ribonucleotide				
mRNA	siControl 1	0,02659	0,06315	0,16688	0,01490	0,00042
mRNA	siDyskerin 1	0,02155	0,06244	0,16906	0,00997	0,00029
mRNA	siControl 2	0,07407	0,06471	0,22540	0,00023	0,00218
mRNA	siDyskerin 2	0,05407	0,06804	0,22074	0,00086	0,00217
mRNA	siControl 3	0,06173	0,05550	0,20982	0,00086	0,00236
mRNA	siDyskerin 3	0,05089	0,07039	0,23504	0,00030	0,00168
mRNA	siControl 4	0,03778	0,03734	0,16290	0,00189	0,00236
mRNA	siDyskerin 4	0,02550	0,04958	0,21756	0,00009	0,00113
Total RNA (mRNA depleted)	siControl 1	5,03495	0,03177	0,14570	0,02206	0,09479
Total RNA (mRNA depleted)	siDyskerin 1	3,38296	0,03016	0,11377	0,02574	0,08379
Total RNA (mRNA depleted)	siControl 2	3,24156	0,03852	0,09880	0,00021	0,09847
Total RNA (mRNA depleted)	siDyskerin 2	2,48631	0,04351	0,09471	0,00049	0,09465
Total RNA (mRNA depleted)	siControl 3	3,38460	0,04769	0,10498	0,00022	0,10849
Total RNA (mRNA depleted)	siDyskerin 3	2,78012	0,04883	0,10246	0,00022	0,10432
Total RNA (mRNA depleted)	siControl 4	3,60827	0,01942	0,07020	0,00028	0,06052
Total RNA (mRNA depleted)	siDyskerin 4	3,34134	0,03374	0,08234	0,00025	0,09529
					tRNA specific	18S rRNA specific

RNA type	Treatment	ψ/C (%)	m7G/C (%)	m6A/C (%)	m2,2G/C (%)	m6,6A/C (%)
		Modification relative to unmodified C				
mRNA	siControl 1	0,02756	0,07891	0,23927	0,01862	0,00060
mRNA	siDyskerin 1	0,02105	0,07883	0,22954	0,01259	0,00039
mRNA	siControl 2	0,08497	0,07401	0,31954	0,00027	0,00310
mRNA	siDyskerin 2	0,06369	0,07802	0,32862	0,00099	0,00324
mRNA	siControl 3	0,07231	0,06626	0,32590	0,00103	0,00366
mRNA	siDyskerin 3	0,05894	0,08074	0,34919	0,00034	0,00250
mRNA	siControl 4	0,04257	0,04487	0,26924	0,00227	0,00391
mRNA	siDyskerin 4	0,03070	0,05623	0,30178	0,00010	0,00156
Total RNA (mRNA depleted)	siControl 1	2,79384	0,03900	0,09074	0,02708	0,05903
Total RNA (mRNA depleted)	siDyskerin 1	1,95788	0,03777	0,07491	0,03223	0,05517
Total RNA (mRNA depleted)	siControl 2	2,07738	0,04402	0,06436	0,00024	0,06414
Total RNA (mRNA depleted)	siDyskerin 2	1,65455	0,04827	0,06292	0,00055	0,06287
Total RNA (mRNA depleted)	siControl 3	2,27944	0,05207	0,06852	0,00024	0,07080
Total RNA (mRNA depleted)	siDyskerin 3	1,88801	0,05388	0,06965	0,00025	0,07092
Total RNA (mRNA depleted)	siControl 4	1,94432	0,02120	0,03758	0,00030	0,03240
Total RNA (mRNA depleted)	siDyskerin 4	2,05664	0,03770	0,05177	0,00028	0,05991
					tRNA specific	18S rRNA specific

Table S2. Cells and culture conditions

Cell Line	Culture Conditions	Supplier
U2OS, HCT116, MCF7, WI-38	DMEM<br>+ 10% FBS<br>+ 1% penicillin/streptomycin	ATCC
U2OS 2-6-3 CLTon	DMEM<br>+ 10% TET-free FBS	Gifted by K. V. Prasanth (University of Illinois)
EBV-transformed lymphoblasts<br>Derived from dyskeratosis congenita patient (NCI-106-1, K314R *DKC1*) and healthy male control	RPMI<br>+ 20% FBS<br>+ 0.01 M HEPES<br>+ 2 mM L-glutamine<br>+ 50 µM/ml gentamycin	Provided by S. A. Savage (NIH) under NIH IRB-approved protocol, ClinicalTrials.gov Identifier: NCT00027274, as described in Niewisch et al. *(36)*
Primary fibroblasts<br>Derived from dyskeratosis congenita patients (GM01774/L37del and AG04646/A386T) and health female control (GM01786, unaffected mother of GM01774)	EMEM with Earle's salts and non-essential amino acids<br>+ 15% FBS	Coriell Cell Repository

Table S3. Oligonucleotides and plasmids

**siRNAs**			
		**Supplier**	**Cat. No**
siControl		Qiagen	1027280
siDyskerin		Dharmacon	L-013639-00
siDyskerin #7		Dharmacon	J-013639-07
siSRRM2		Dharmacon	L-015368-00
siGAR1		Dharmacon	L-013386-00
siSRSF2		Dharmacon	L-019711-00
**Plasmids**			
mEGFP-23		Cloned in house from Addgene #56463 (H4 removed)	
mCherry-23		Cloned in house from Addgene #55058 (H3 removed)	
pCMV-T7-EGFP		Addgene #133962	
**qPCR primers**			
		**Sequence**	**Source**
ACTB -72	Forward	CCGAAAGTTGCCTTTTATGGC	(*80*)
	Reverse	CAAAGGCGAGGCTCTGTGC	(*80*)
ACTB 332	Forward	CGGGGTCTTTGTCTGAGC	(*80*)
	Reverse	CAGTTAGCGCCCAAAGGAC	(*80*)
ACTB 3752	Forward	GGGACTATTTGGGGGTGTCT	(*80*)
	Reverse	TCCCATAGGTGAAGGCAAAG	(*80*)
ACTB	Forward	AGAGCTACGAGCTGCCTGAC	Own design
	Reverse	AGCACTGTGTTGGCGTACAG	Own design
EEF2	Forward	AAACTGGACAGCGAGGACAA	Own design
	Reverse	TGGGGTCACAGCTTTTAATGC	Own design
SRRM2	Forward	GTCATTCTGGGTCCTCCTCA	Own design
	Reverse	CTCTTGGGGACATGGTGACT	Own design
45S	Forward	CTCCGTTATGGTAGCGCTGC	Own design
	Reverse	GCGGAACCCTCGCTTCTC	Own design
snU2	Forward	GGCCTTTTGGCTAAGATCAAG	Own design
	Reverse	GGAGCAAGCTCCTATTCCATC	Own design
Rabbit beta-globin	Forward	GTTCATTAGATCCTGAGAACTTCAG	(*55*)
	Reverse	AAAGATCTCAGTGGTATTTGTGAGC	(*55*)
GFP	Forward	ACGAGGGCAA TACAAGACC	Own design
	Reverse	TTGTACTCCAGCTTGTGCCC	Own design
mCherry	Forward	CACTACGACGCTGAGGTCAA	Own design
	Reverse	GTAGTCCTCGTTGTGGGAGG	Own design
45S	Forward	GAACGGTGGTGTGTCGTT	(*81*)
	Reverse	GCTTCTCGTCTCGTCTCACT	
18S 5' Junction	Forward	GCCGCGCTCTACCTTACCTACCT	(*81*)
	Reverse	CAGACATGCATGGCTTAATCTTTG	
Mature ***18S***	Forward	GATGGTAGTCGCCGTGCC	(*81*)
	Reverse	GCCTGCTGCCTTCCTTGG	
18S 3' Junction	Forward	AGTCGTAACAAGGTTTCCGTAGGT	(*81*)
	Reverse	CCTCCGGGCTCCGTTAAT	
5.8S 5' junction	Forward	TACGACTCTTAGCGGTGGATCA	(*81*)
	Reverse	TCACATTAATTCTCGCAGCTAGCT	
Mature ***5.8S***	Forward	ACTCGGCTCGTGCGTC	(*81*)
	Reverse	GCGACGCTCAGACAGG	
5.8S 3' Junction	Forward	GAATTGCAGGACACATTAATCATC	(*81*)
	Reverse	GGCAAGCGACGCTCAGA	
	Forward	CCGAGACGCGACCTCAGAT	(*81*)

28S 5' Junction	Reverse	TCCGCTGACTAATATGCTTAAATTCA	
Mature ***28S***	Forward	GTGACGGCGATGAATGGA	(*81*)
	Reverse	TGTGGTTTCGCTGGATAGTAGGT	
**Plasmid primers**			
		**Sequence**	**Source**
CMV Forward		CGCAAATGGGCGGTAGG	Common sequencing primer
BGH Reverse		AACTAGAAGGCACAGTCG	Common sequencing primer

Table S4. Antibodies

Antibody	Catalogue No	Supplier	Species	Used for
Coilin	sc-32860	Santa Cruz Biotechnologies	Mouse	IF
Dyskerin	H00001736-m04	Abnova	Mouse	IF, IP, ChIP
Dyskerin	HPA001022	Atlas antibodies	Rabbit	IF, WB
Dyskerin	sc-48794	Santa Cruz Biotechnologies	Rabbit	WB
GAR1	11711-1-AP	Proteintech	Rabbit	IF, WB, IP
GFP	ab290	Abcam	Rabbit	WB
mCherry	ab167453	Abcam	Rabbit	WB
mouse IgG	12-371	Merck Millipore	Mouse	IP
NHP2	ab204352	Abcam	Rabbit	IF, WB
NHP2	sc398430	Santa Cruz Biotechnologies	Mouse	WB
NOP10	ab134902	Abcam	Rabbit	IF, WB
Nucleolin	ab22758	Abcam	Rabbit	IF
Pseudouridine	D347-3	MBL	Mouse	IP
Puromycin	MABE343	Merck Millipore	Mouse	WB
RNAPII 8WG16	ab817	Abcam	Mouse	WB, ChIP-qPCR
RNAPII CTD	MAB0601	MBL	Mouse	ChIP-seq
RNAPII S2P	MABI0602	MBL	Mouse	WB
RNAPII S5P	ab5408	Abcam	Mouse	WB, IP
SC-35	ab39578	Abcam	Mouse	IF
SF3B1	ab39578	Abcam	Rabbit	IF
SRRM2	NBP2-55697	Novus	Rabbit	IF
β-actin	A5441	Sigma Aldrich	Mouse	WB
**Secondary Antibody**	**Catalogue No**	**Supplier**	**Species**	**Used for**
Goat anti-Mouse IgG, AlexaFluor 488	A-11029	Thermo Fisher Scientific	goat	IF
Donkey anti-Mouse IgG, AlexaFluor 594	A-21203	Thermo Fisher Scientific	donkey	IF
Goat anti-Rabbit IgG, AlexaFluor 488	A-11008	Thermo Fisher Scientific	goat	IF
Donkey anti-Rabbit IgG, AlexaFluor 594	A-21207	Thermo Fisher Scientific	donkey	IF
Anti-mouse IgG, HRP-Linked	7076	Cell Signaling	Horse	WB
Anti-rabbit IgG, HRP-Linked	7074	Cell Signaling	Goat	WB

Table S5. Software and algorithms

	Source	Availability
**GraphPad Prism 9.1.0**	GraphPad	https://www.graphpad.com/scientific-software/prism/
**Inkscape 1.2.1 (for figure preparation)**	Inkscape	https://inkscape.org/release/inkscape-1.2.1/
**CellProfiler 3.1.9**	(*82*)	https://cellprofiler.org/
**Fiji 2.3.0**	(*83*)	https://fiji.sc/
**R 4.1.1**	R Core Team (2021). R: A language and environment for statistical computing. R Foundation for Statistical Computing, Vienna, Austria	https://www.r-project.org/
**RStudio**	RStudio	https://www.rstudio.com/
**FastQC 0.11.9**	https://www.bioinformatics.babraham.ac.uk/projects/fastqc/	https://github.com/s-andrews/FastQC
**TrimGalore 6.0.4_dev**	https://www.bioinformatics.babraham.ac.uk/projects/trim_galore/	https://github.com/FelixKrueger/TrimGalore
**bwa 0.7.17 (r1188)**	https://arxiv.org/abs/1303.3997	https://github.com/lh3/bwa
**Bowtie 2.4.1**	(*84*)	http://bowtie-bio.sourceforge.net/bowtie2/index.shtml
**STAR 2.7.0e**	(*85*)	https://github.com/alexdobin/STAR/releases
**Picard MarkDuplicates 2.18.11**	https://broadinstitute.github.io/picard/	https://github.com/broadinstitute/picard
**deepTools 3.2.1**	(*86*)	https://github.com/deeptools/deepTools/
**Salmon 1.2.0**	(*87*)	https://github.com/COMBINE-lab/salmon
**tximport**	(*88*)	https://bioconductor.org/packages/release/bioc/html/tximport.html
**DESeq2**	(*89*)	https://bioconductor.org/packages/release/bioc/html/DESeq2.html
**IHW**	(*90*)	https://bioconductor.org/packages/release/bioc/html/IHW.html
**rMATS 4.1.2**	(*91*)	http://rnaseq-mats.sourceforge.net/
**iMaps**		https://imaps.goodwright.com/
**Cutadapt**	(*92*)	https://cutadapt.readthedocs.io/en/stable/
**iCount 2.0.1**		https://github.com/tomazc/iCount
**ggplot2**	(*93*)	https://ggplot2.tidyverse.org/

REFERENCES AND NOTES

1. F. F. Davis, F. W. Allen, Ribonucleic acids from yeast which contain a fifth nucleotide. *J. Biol. Chem.* **227**, 907–915 (1957).

2. W. E. Cohn, E. Volkin, Nucleoside-5′-phosphates from ribonucleic acid. *Nature* **167**, 483–484 (1951).

3. E. K. Borchardt, N. M. Martinez, W. V. Gilbert, Regulation and function of RNA pseudouridylation in human cells. *Annu. Rev. Genet.* **54**, 309–336 (2020).

4. J. Ge, Y.-T. Yu, RNA pseudouridylation: New insights into an old modification. *Trends Biochem. Sci.* **38**, 210–218 (2013).

5. T. M. Carlile, M. F. Rojas-Duran, B. Zinshteyn, H. Shin, K. M. Bartoli, W. V. Gilbert, Pseudouridine profiling reveals regulated mRNA pseudouridylation in yeast and human cells. *Nature* **515**, 143–146 (2014).

6. S. Schwartz, D. A. Bernstein, M. R. Mumbach, M. Jovanovic, R. H. Herbst, B. X. León-Ricardo, J. M. Engreitz, M. Guttman, R. Satija, E. S. Lander, G. Fink, A. Regev, Transcriptome-wide mapping reveals widespread dynamic-regulated pseudouridylation of ncRNA and mRNA. *Cell* **159**, 148–162 (2014).

7. V. Khoddami, A. Yerra, T. L. Mosbruger, A. M. Fleming, C. J. Burrows, B. R. Cairns, Transcriptome-wide profiling of multiple RNA modifications simultaneously at single-base resolution. *Proc. Natl. Acad. Sci. U.S.A.* **116**, 6784–6789 (2019).

8. A. F. Lovejoy, D. P. Riordan, P. O. Brown, Transcriptome-wide mapping of pseudouridines: pseudouridine synthases modify specific mRNAs in S. cerevisiae. *PLOS ONE* **9**, e110799 (2014).

9. X. Li, P. Zhu, S. Ma, J. Song, J. Bai, F. Sun, C. Yi, Chemical pulldown reveals dynamic pseudouridylation of the mammalian transcriptome. *Nat. Chem. Biol.* **11**, 592–597 (2015).

10. Q. Dai, L.-S. Zhang, H.-L. Sun, K. Pajdzik, L. Yang, C. Ye, C.-W. Ju, S. Liu, Y. Wang, Z. Zheng, L. Zhang, B. T. Harada, X. Dou, I. Irkliyenko, X. Feng, W. Zhang, T. Pan, C. He, Quantitative sequencing using BID-seq uncovers abundant pseudouridines in mammalian mRNA at base resolution. *Nat. Biotechnol.* **41**, 344–354 (2023).

11. G. Kan, Z. Wang, C. Sheng, G. Chen, C. Yao, Y. Mao, S. Chen, Dual inhibition of DKC1 and MEK1/2 synergistically restrains the growth of colorectal cancer cells. *Adv. Sci. (Weinh)* **8**, 2004344 (2021).

12. B. R. Anderson, H. Muramatsu, B. K. Jha, R. H. Silverman, D. Weissman, K. Kariko, Nucleoside modifications in RNA limit activation of 2'-5'-oligoadenylate synthetase and increase resistance to cleavage by RNase L. *Nucleic Acids Res.* **39**, 9329–9338 (2011).

13. K. Leppek, G. W. Byeon, W. Kladwang, H. K. Wayment-Steele, C. H. Kerr, A. F. Xu, D. S. Kim, V. V. Topkar, C. Choe, D. Rothschild, G. C. Tiu, R. Wellington-Oguri, K. Fujii, E. Sharma, A. M. Watkins, J. J. Nicol, J. Romano, B. Tunguz, F. Diaz, H. Cai, P. Guo, J. Wu, F. Meng, S. Shi, E. Participants, P. R. Dormitzer, A. Solórzano, M. Barna, R. das, Combinatorial optimization of mRNA structure, stability, and translation for RNA-based therapeutics. *Nat. Commun.* **13**, 1536 (2022).

14. D. E. Eyler, M. K. Franco, Z. Batool, M. Z. Wu, M. L. Dubuke, M. Dobosz-Bartoszek, J. D. Jones, Y. S. Polikanov, B. Roy, K. S. Koutmou, Pseudouridinylation of mRNA coding sequences alters translation. *Proc. Natl. Acad. Sci. U.S.A.* **116**, 23068–23074 (2019).

15. K. Karikó, H. Muramatsu, F. A. Welsh, J. Ludwig, H. Kato, S. Akira, D. Weissman, Incorporation of pseudouridine into mRNA yields superior nonimmunogenic vector with increased translational capacity and biological stability. *Mol. Ther.* **16**, 1833–1840 (2008).

16. J. Karijolich, Y.-T. Yu, Converting nonsense codons into sense codons by targeted pseudouridylation. *Nature* **474**, 395–398 (2011).

17. Y. V. Svitkin, A.-C. Gingras, N. Sonenberg, Membrane-dependent relief of translation elongation arrest on pseudouridine- and N1-methyl-pseudouridine-modified mRNAs. *Nucleic Acids Res.* **50**, 7202–7215 (2022).

18. Y. V. Svitkin, Y. M. Cheng, T. Chakraborty, V. Presnyak, M. John, N. Sonenberg, N1-methyl-pseudouridine in mRNA enhances translation through eIF2α-dependent and independent mechanisms by increasing ribosome density. *Nucleic Acids Res.* **45**, 6023–6036 (2017).

19. B. R. Anderson, H. Muramatsu, S. R. Nallagatla, P. C. Bevilacqua, L. H. Sansing, D. Weissman, K. Karikó, Incorporation of pseudouridine into mRNA enhances translation by diminishing PKR activation. *Nucleic Acids Res.* **38**, 5884–5892 (2010).

20. C. Chen, X. Zhao, R. Kierzek, Y.-T. Yu, A flexible RNA backbone within the polypyrimidine tract is required for U2AF65 binding and pre-mRNA splicing in vivo. *Mol. Cell. Biol.* **30**, 4108–4119 (2010).

21. N. M. Martinez, A. Su, M. C. Burns, J. K. Nussbacher, C. Schaening, S. Sathe, G. W. Yeo, W. V. Gilbert, Pseudouridine synthases modify human pre-mRNA co-transcriptionally and affect pre-mRNA processing. *Mol. Cell* **82**, 645–659.e9 (2022).

22. C. Martinez Campos, K. Tsai, D. G. Courtney, H. P. Bogerd, C. L. Holley, B. R. Cullen, Mapping of pseudouridine residues on cellular and viral transcripts using a novel antibody-based technique. *RNA* **27**, 1400–1411 (2021).

23. A. Henras, Y. Henry, C. Bousquet-Antonelli, J. Noaillac-Depeyre, J.P. Gélugne, M. Caizergues-Ferrer, Nhp2p and Nop10p are essential for the function of H/ACA snoRNPs. *EMBO J.* **17**, 7078–7090 (1998).

24. D. P. Czekay, U. Kothe, H/ACA small ribonucleoproteins: Structural and functional comparison between archaea and eukaryotes. *Front. Microbiol.* **12**, 654370 (2021).

25. U. T. Meier, The many facets of H/ACA ribonucleoproteins. *Chromosoma* **114**, 1–14 (2005).

26. D. E. MacNeil, P. Lambert-Lanteigne, C. Autexier, N-terminal residues of human dyskerin are required for interactions with telomerase RNA that prevent RNA degradation. *Nucleic Acids Res.* **47**, 5368–5380 (2019).

27. G. E. Ghanim, A. J. Fountain, A.M. M. van Roon, R. Rangan, R. das, K. Collins, T. H. D. Nguyen, Structure of human telomerase holoenzyme with bound telomeric DNA. *Nature* **593**, 449–453 (2021).

28. B. E. Jády, A. Ketele, T. Kiss, Human intron-encoded Alu RNAs are processed and packaged into Wdr79-associated nucleoplasmic box H/ACA RNPs. *Genes Dev.* **26**, 1897–1910 (2012).

29. H. Jorjani, S. Kehr, D. J. Jedlinski, R. Gumienny, J. Hertel, P. F. Stadler, M. Zavolan, A. R. Gruber, An updated human snoRNAome. *Nucleic Acids Res.* **44**, 5068–5082 (2016).

30. N. S. Heiss, S. W. Knight, T. J. Vulliamy, S. M. Klauck, S. Wiemann, P. J. Mason, A. Poustka, I. Dokal, X-linked dyskeratosis congenita is caused by mutations in a highly conserved gene with putative nucleolar functions. *Nat. Genet.* **19**, 32–38 (1998).

31. J. R. Mitchell, E. Wood, K. Collins, A telomerase component is defective in the human disease dyskeratosis congenita. *Nature* **402**, 551–555 (1999).

32. S. W. Knight, N. S. Heiss, T. J. Vulliamy, C. M. Aalfs, C. McMahon, P. Richmond, A. Jones, R. C. M. Hennekam, A. Poustka, P. J. Mason, I. Dokal, Unexplained aplastic anaemia, immunodeficiency, and cerebellar hypoplasia (Hoyeraal-Hreidarsson syndrome) due to mutations in the dyskeratosis congenita gene, DKC1. *Br. J. Haematol.* **107**, 335–339 (1999).

33. A. J. Walne, T. Vulliamy, A. Marrone, R. Beswick, M. Kirwan, Y. Masunari, F.H. al-Qurashi, M. Aljurf, I. Dokal, Genetic heterogeneity in autosomal recessive dyskeratosis congenita with one subtype due to mutations in the telomerase-associated protein NOP10. *Hum. Mol. Genet.* **16**, 1619–1629 (2007).

34. T. Vulliamy, R. Beswick, M. Kirwan, A. Marrone, M. Digweed, A. Walne, I. Dokal, Mutations in the telomerase component NHP2 cause the premature ageing syndrome dyskeratosis congenita. *Proc. Natl. Acad. Sci. U.S.A.* **105**, 8073–8078 (2008).

35. P. Revy, C. Kannengiesser, A. A. Bertuch, Genetics of human telomere biology disorders. *Nat. Rev. Genet.* **24**, 86–108 (2023).

36. M. R. Niewisch, N. Giri, L. J. McReynolds, R. Alsaggaf, S. Bhala, B. P. Alter, S. A. Savage, Disease progression and clinical outcomes in telomere biology disorders. *Blood* **139**, 1807–1819 (2022).

37. I. Dokal, Dyskeratosis congenita in all its forms. *Br. J. Haematol.* **110**, 768–779 (2000).

38. A. Yoon, G. Peng, Y. Brandenburg, O. Zollo, W. Xu, E. Rego, D. Ruggero, Impaired control of IRES-mediated translation in X-linked dyskeratosis congenita. *Science* **312**, 902–906 (2006).

39. E. Balogh, J. C. Chandler, M. Varga, M. Tahoun, D. K. Menyhárd, G. Schay, T. Goncalves, R. Hamar, R. Légrádi, Á. Szekeres, O. Gribouval, R. Kleta, H. Stanescu, D. Bockenhauer, A. Kerti, H. Williams, V. Kinsler, W.L. di, D. Curtis, M. Kolatsi-Joannou, H. Hammid, A. Szőcs, K. Perczel, E. Maka, G. Toldi, F. Sava, C. Arrondel, M. Kardos, A. Fintha, A. Hossain, F. D'Arco, M. Kaliakatsos, J. Koeglmeier, W. Mifsud, M. Moosajee, A. Faro, E. Jávorszky, G. Rudas, M. H. Saied, S. Marzouk, K. Kelen, J. Götze, G. Reusz, T. Tulassay, F. Dragon, G. Mollet, S. Motameny, H. Thiele, G. Dorval, P. Nürnberg, A. Perczel, A. J. Szabó, D. A. Long, K. Tomita, C. Antignac, A. M. Waters, K. Tory, Pseudouridylation defect due to DKC1 and NOP10 mutations causes nephrotic syndrome with cataracts, hearing impairment, and enterocolitis. *Proc. Natl. Acad. Sci. U.S.A.* **117**, 15137–15147 (2020).

40. J. He, S. Navarrete, M. Jasinski, T. Vulliamy, I. Dokal, M. Bessler, P. J. Mason, Targeted disruption of Dkc1, the gene mutated in X-linked dyskeratosis congenita, causes embryonic lethality in mice. *Oncogene* **21**, 7740–7744 (2002).

41. M. C. Carneiro, C. M. Henriques, J. Nabais, T. Ferreira, T. Carvalho, M. G. Ferreira, Short telomeres in key tissues initiate local and systemic aging in zebrafish. *PLOS Genet.* **12**, e1005798 (2016).

42. T. C. Pereboom, L. J. van Weele, A. Bondt, A. W. MacInnes, A zebrafish model of dyskeratosis congenita reveals hematopoietic stem cell formation failure resulting from ribosomal protein-mediated p53 stabilization. *Blood* **118**, 5458–5465 (2011).

43. A. I. Lamond, D. L. Spector, Nuclear speckles: A model for nuclear organelles. *Nat. Rev. Mol. Cell Biol.* **4**, 605–612 (2003).

44. X. Wei, S. Somanathan, J. Samarabandu, R. Berezney, Three-dimensional visualization of transcription sites and their association with splicing factor-rich nuclear speckles. *J. Cell Biol.* **146**, 543–558 (1999).

45. L. Galganski, M. O. Urbanek, W. J. Krzyzosiak, Nuclear speckles: molecular organization, biological function and role in disease. *Nucleic Acids Res.* **45**, 10350–10368 (2017).

46. S. Hu, P. Lv, Z. Yan, B. Wen, Disruption of nuclear speckles reduces chromatin interactions in active compartments. *Epigenetics Chromatin* **12**, 43 (2019).

47. J. Fei, M. Jadaliha, T.S. Harmon, I.T.S. Li, B. Hua, Q. Hao, A.S. Holehouse, M. Reyer, Q. Sun, S.M. Freier, R.V. Pappu, K.V. Prasanth, T. Ha, Quantitative analysis of multilayer organization of proteins and RNA in nuclear speckles at super resolution. *J. Cell Sci.* **130**, 4180–4192 (2017).

48. X. Darzacq, N. Kittur, S. Roy, Y. Shav-Tal, R. H. Singer, U. T. Meier, Stepwise RNP assembly at the site of H/ACA RNA transcription in human cells. *J. Cell Biol.* **173**, 207–218 (2006).

49. F. Alawi, P. Lin, Dyskerin is required for tumor cell growth through mechanisms that are independent of its role in telomerase and only partially related to its function in precursor rRNA processing. *Mol. Carcinog.* **50**, 334–345 (2011).

50. I. Huppertz, J. Attig, A. D'Ambrogio, L. E. Easton, C. R. Sibley, Y. Sugimoto, M. Tajnik, J. König, J. Ule, iCLIP: protein-RNA interactions at nucleotide resolution. *Methods* **65**, 274–287 (2014).

51. N. Viphakone, I. Sudbery, L. Griffith, C. G. Heath, D. Sims, S. A. Wilson, Co-transcriptional loading of RNA export factors shapes the human transcriptome. *Mol. Cell* **75**, 310–323.e8 (2019).

52. C. Bellodi, M. McMahon, A. Contreras, D. Juliano, N. Kopmar, T. Nakamura, D. Maltby, A. Burlingame, S. A. Savage, A. Shimamura, D. Ruggero, H/ACA small RNA dysfunctions in disease reveal key roles for noncoding RNA modifications in hematopoietic stem cell differentiation. *Cell Rep.* **3**, 1493–1502 (2013).

53. M. Taoka, Y. Nobe, Y. Yamaki, K. Sato, H. Ishikawa, K. Izumikawa, Y. Yamauchi, K. Hirota, H. Nakayama, N. Takahashi, T. Isobe, Landscape of the complete RNA chemical modifications in the human 80S ribosome. *Nucleic Acids Res.* **46**, 9289–9298 (2018).

54. S. M. Janicki, T. Tsukamoto, S. E. Salghetti, W. P. Tansey, R. Sachidanandam, K. V. Prasanth, T. Ried, Y. Shav-Tal, E. Bertrand, R. H. Singer, D. L. Spector, From silencing to gene expression: Real-time analysis in single cells. *Cell* **116**, 683–698 (2004).

55. V. Tripathi, D. Y. Song, X. Zong, S. P. Shevtsov, S. Hearn, X.D. Fu, M. Dundr, K. V. Prasanth, SRSF1 regulates the assembly of pre-mRNA processing factors in nuclear speckles. *Mol. Biol. Cell* **23**, 3694–3706 (2012).

56. D. L. Baker, O. A. Youssef, M. I.R. Chastkofsky, D. A. Dy, R. M. Terns, M. P. Terns, RNA-guided RNA modification: Functional organization of the archaeal H/ACA RNP. *Genes Dev.* **19**, 1238–1248 (2005).

57. F. Zacchini, G. Venturi, V. de Sanctis, R. Bertorelli, C. Ceccarelli, D. Santini, M. Taffurelli, M. Penzo, D. Treré, A. Inga, E. Dassi, L. Montanaro, Human dyskerin binds to cytoplasmic H/ACA-box-containing transcripts affecting nuclear hormone receptor dependence. *Genome Biol.* **23**, 177 (2022).

58. J. Ge, D. A. Rudnick, J. He, D. L. Crimmins, J. H. Ladenson, M. Bessler, P. J. Mason, Dyskerin ablation in mouse liver inhibits rRNA processing and cell division. *Mol. Cell. Biol.* **30**, 413–422 (2010).

59. Y. Mochizuki, J. He, S. Kulkarni, M. Bessler, P. J. Mason, Mouse dyskerin mutations affect accumulation of telomerase RNA and small nucleolar RNA, telomerase activity, and ribosomal RNA processing. *Proc. Natl. Acad. Sci. U.S.A.* **101**, 10756–10761 (2004).

60. K. Nishimura, T. Kumazawa, T. Kuroda, N. Katagiri, M. Tsuchiya, N. Goto, R. Furumai, A. Murayama, J. Yanagisawa, K. Kimura, Perturbation of ribosome biogenesis drives cells into senescence through 5S RNP-mediated p53 activation. *Cell Rep.* **10**, 1310–1323 (2015).

61. L. Tafforeau, C. Zorbas, J.-L. Langhendries, S.-T. Mullineux, V. Stamatopoulou, R. Mullier, L. Wacheul, D. L. J. Lafontaine, The complexity of human ribosome biogenesis revealed by systematic nucleolar screening of pre-rRNA processing factors. *Mol. Cell* **51**, 539–551 (2013).

62. K. Jack, C. Bellodi, D. M. Landry, R. O. Niederer, A. Meskauskas, S. Musalgaonkar, N. Kopmar, O. Krasnykh, A. M. Dean, S. R. Thompson, D. Ruggero, J. D. Dinman, rRNA pseudouridylation defects affect ribosomal ligand binding and translational fidelity from yeast to human cells. *Mol. Cell* **44**, 660–666 (2011).

63. J. Baßler, E. Hurt, Eukaryotic ribosome assembly. *Annu. Rev. Biochem.* **88**, 281–306 (2019).

64. R. Nir, T. P. Hoernes, H. Muramatsu, K. Faserl, K. Karikó, M. D. Erlacher, A. Sas-Chen, S. Schwartz, A systematic dissection of determinants and consequences of snoRNA-guided pseudouridylation of human mRNA. *Nucleic Acids Res.* **50**, 4900–4916 (2022).

65. A. Garus, C. Autexier, Dyskerin: An essential pseudouridine synthase with multifaceted roles in ribosome biogenesis, splicing, and telomere maintenance. *RNA* **27**, 1441–1458 (2021).

66. G. Wu, M. Xiao, C. Yang, Y.-T. Yu, U2 snRNA is inducibly pseudouridylated at novel sites by Pus7p and snR81 RNP. *EMBO J.* **30**, 79–89 (2011).

67. E. A. Caton, E. K. Kelly, R. Kamalampeta, U. Kothe, Efficient RNA pseudouridylation by eukaryotic H/ACA ribonucleoproteins requires high affinity binding and correct positioning of guide RNA. *Nucleic Acids Res.* **46**, 905–916 (2018).

68. M. D. De Zoysa, G. Wu, R. Katz, Y.-T. Yu, Guide-substrate base-pairing requirement for box H/ACA RNA-guided RNA pseudouridylation. *RNA* **24**, 1106–1117 (2018).

69. E. K. Kelly, D. P. Czekay, U. Kothe, Base-pairing interactions between substrate RNA and H/ACA guide RNA modulate the kinetics of pseudouridylation, but not the affinity of substrate binding by H/ACA small nucleolar ribonucleoproteins. *RNA* **25**, 1393–1404 (2019).

70. L. L. Hall, D. M. Carone, A. V. Gomez, H. J. Kolpa, M. Byron, N. Mehta, F. O. Fackelmayer, J. B. Lawrence, Stable C0T-1 repeat RNA is abundant and is associated with euchromatic interphase chromosomes. *Cell* **156**, 907–919 (2014).

71. A. Ketele, T. Kiss, B. E. Jády, Human intron-encoded AluACA RNAs and telomerase RNA share a common element promoting RNA accumulation. *RNA Biol.* **13**, 1274–1285 (2016).

72. B. Roy, Effects of mRNA Modifications on Translation: An Overview. *Methods Mol. Biol.* **2298**, 327–356 (2021).

73. T. P. Hoernes, N. Clementi, K. Faserl, H. Glasner, K. Breuker, H. Lindner, A. Hüttenhofer, M. D. Erlacher, Nucleotide modifications within bacterial messenger RNAs regulate their translation and are able to rewire the genetic code. *Nucleic Acids Res.* **44**, 852–862 (2016).

74. S. R. Nallagatla, P. C. Bevilacqua, Nucleoside modifications modulate activation of the protein kinase PKR in an RNA structure-specific manner. *RNA* **14**, 1201–1213 (2008).

75. O. A. Youssef, S. A. Safran, T. Nakamura, D. A. Nix, G. S. Hotamisligil, B. L. Bass, Potential role for snoRNAs in PKR activation during metabolic stress. *Proc. Natl. Acad. Sci. U.S.A.* **112**, 5023–5028 (2015).

76. C. Bellodi, N. Kopmar, D. Ruggero, Deregulation of oncogene-induced senescence and p53 translational control in X-linked dyskeratosis congenita. *EMBO J.* **29**, 1865–1876 (2010).

77. M. Hallegger, A. M. Chakrabarti, F. C.Y. Lee, B. L. Lee, A. G. Amalietti, H. M. Odeh, K. E. Copley, J. D. Rubien, B. Portz, K. Kuret, I. Huppertz, F. Rau, R. Patani, N. L. Fawzi, J.

Shorter, N. M. Luscombe, J. Ule, TDP-43 condensation properties specify its RNA-binding and regulatory repertoire. *Cell* **184**, 4680–4696.e22 (2021).

78. J. Ristau, K. Watt, C. Oertlin, O. Larsson, Polysome fractionation for transcriptome-wide studies of mRNA translation. *Methods Mol. Biol.* **2418**, 223–241 (2022).

79. L. Zhang, Y. Zhang, Y. Chen, O. Gholamalamdari, Y. Wang, J. Ma, A. S. Belmont, TSA-seq reveals a largely conserved genome organization relative to nuclear speckles with small position changes tightly correlated with gene expression changes. *Genome Res.* **31**, 251–264 (2020).

80. D. Y. Zhao, G. Gish, U. Braunschweig, Y. Li, Z. Ni, F. W. Schmitges, G. Zhong, K. Liu, W. Li, J. Moffat, M. Vedadi, J. Min, T. J. Pawson, B. J. Blencowe, J. F. Greenblatt, SMN and symmetric arginine dimethylation of RNA polymerase II C-terminal domain control termination. *Nature* **529**, 48–53 (2016).

81. I. Kwon, S. Xiang, M. Kato, L. Wu, P. Theodoropoulos, T. Wang, J. Kim, J. Yun, Y. Xie, S. L. McKnight, Poly-dipeptides encoded by the C9orf72 repeats bind nucleoli, impede RNA biogenesis, and kill cells. *Science* **345**, 1139–1145 (2014).

82. C. McQuin, A. Goodman, V. Chernyshev, L. Kamentsky, B. A. Cimini, K. W. Karhohs, M. Doan, L. Ding, S. M. Rafelski, D. Thirstrup, W. Wiegraebe, S. Singh, T. Becker, J. C. Caicedo, A. E. Carpenter, CellProfiler 3.0: Next-generation image processing for biology. *PLOS Biol.* **16**, e2005970 (2018).

83. J. Schindelin, I. Arganda-Carreras, E. Frise, V. Kaynig, M. Longair, T. Pietzsch, S. Preibisch, C. Rueden, S. Saalfeld, B. Schmid, J. Y. Tinevez, D. J. White, V. Hartenstein, K. Eliceiri, P. Tomancak, A. Cardona, Fiji: An open-source platform for biological-image analysis. *Nat. Methods* **9**, 676–682 (2012).

84. B. Langmead, S. L. Salzberg, Fast gapped-read alignment with Bowtie 2. *Nat. Methods* **9**, 357–359 (2012).

85. A. Dobin, C. A. Davis, F. Schlesinger, J. Drenkow, C. Zaleski, S. Jha, P. Batut, M. Chaisson, T. R. Gingeras, STAR: Ultrafast universal RNA-seq aligner. *Bioinformatics* **29**, 15–21 (2013).

86. F. Ramírez, F. Dündar, S. Diehl, B. A. Grüning, T. Manke, deepTools: A flexible platform for exploring deep-sequencing data. *Nucleic Acids Res.* **42**, W187–W191 (2014).

87. R. Patro, G. Duggal, M. I. Love, R. A. Irizarry, C. Kingsford, Salmon provides fast and bias-aware quantification of transcript expression. *Nat. Methods* **14**, 417–419 (2017).

88. C. Soneson, M. I. Love, M. D. Robinson, Differential analyses for RNA-seq: transcript-level estimates improve gene-level inferences. *F1000Res* **4**, 1521 (2015).

89. M. I. Love, W. Huber, S. Anders, Moderated estimation of fold change and dispersion for RNA-seq data with DESeq2. *Genome Biol.* **15**, 550 (2014).

90. N. Ignatiadis, B. Klaus, J. B. Zaugg, W. Huber, Data-driven hypothesis weighting increases detection power in genome-scale multiple testing. *Nat. Methods* **13**, 577–580 (2016).

91. S. Shen, J. W. Park, Z. X. Lu, L. Lin, M. D. Henry, Y. N. Wu, Q. Zhou, Y. Xing, rMATS: Robust and flexible detection of differential alternative splicing from replicate RNA-Seq data. *Proc. Natl. Acad. Sci. U.S.A.* **111**, E5593–E5601 (2014).

92. T. Magoč, S. L. Salzberg, FLASH: Fast length adjustment of short reads to improve genome assemblies. *Bioinformatics* **27**, 2957–2963 (2011).

93. F. Pedregosa, G. Varoquaux, A. Gramfort, V. Michel, B. Thirion, O. Grisel, M. Blondel, P. Prettenhofer, R. Weiss, V. Dubourg, J. Vanderplas, A. Passos, D. Cournapeau, M. Brucher, M. Perrot, É. Duchesna, Scikit-learn: Machine learning in Python. *J. Mach. Learn. Res.* **12**, 2825–2830 (2011).
